# Comparative Efficacy of Medical Treatments for Chronic Heart Failure: A Network Meta-Analysis

**DOI:** 10.3389/fcvm.2021.787810

**Published:** 2022-01-13

**Authors:** Boyang Xiang, Zongliang Yu, Xiang Zhou

**Affiliations:** ^1^Department of Cardiology, The Second Affiliated Hospital of Soochow University, Suzhou, China; ^2^Department of Cardiology, The First People's Hospital of Kunshan Affiliated to Jiangsu University, Kunshan, China

**Keywords:** chronic heart failure, medical treatment, meta-analysis, hospitalization, mortality

## Abstract

**Background:** The medical treatments of chronic heart failure have made remarkable progress in recent years. It is crucial to determine the optimal drug combination based on current evidence.

**Methods:** A search of PubMed, EMBASE, and Cochrane CENTRAL databases was conducted for studies on angiotensin receptor-neprilysin inhibitors (ARNIs), sodium-glucose cotransporter 2 inhibitors (SGLT2is), angiotensin-converting enzyme inhibitors (ACEIs), angiotensin receptor blockers (ARBs), beta-blockers (BBs), mineralocorticoid receptor antagonists (MRAs), and ivabradine (IVA) between 1987 and 2021. The network meta-analysis was performed to compare the efficacy of drug therapies in heart failure with reduced ejection fraction (HFrEF).

**Results:** Forty-eight randomized controlled trials (RCTs), which overall included 68,074 patients with HF and left ventricular ejection fraction (LVEF) ≤ 40%, were identified and included in the network meta-analysis. The efficacies of 13 intervention classes, including monotherapies or combinations of ACEI, ARB, ARNI, BB, MRA, SGLT2i, IVA, and placebo, on hospitalization for HF, cardiovascular mortality, and all-cause mortality were compared. Among the 13 included interventions, ARNI+BB+MRA, SGLT2i+ACEI+BB+MRA, and IVA+ACEI+BB+MRA were found to be best in terms of all three outcomes. Compared with placebo, these three drug combinations were associated with significant reductions in the risk of all-cause death, cardiovascular mortality and hospitalization for HF.

**Conclusions:** ARNI+BB+MRA, SGLT2i+ACEI+BB+MRA, and IVA+ACEI+BB+MRA were the top three therapies for patients with HFrEF. The increasing use of combinations of conventional and novel drugs contributed to progressive reductions in hospitalization and mortality in patients with HFrEF.

## Introduction

Heart failure (HF), a complex clinical syndrome with significant morbidity and mortality, currently affects more than 26 million people worldwide and is rapidly escalating in prevalence. Approximately half of all HF patients have heart failure with reduced ejection fraction (HFrEF) (1–3). Conventional treatments for HFrEF include angiotensin-converting enzyme inhibitors (ACEIs), angiotensin receptor blockers (ARBs), mineralocorticoid receptor antagonists (MRAs), and beta-blockers (BBs). With continuous breakthroughs in drug therapies, the optimal treatment for HFrEF continues to be redefined. Many novel drugs, such as angiotensin receptor-neprilysin inhibitors (ARNIs), sodium-glucose cotransporter 2 inhibitors (SGLT2is), and ivabradine (IVA), have been recommended for the treatment of patients with HFrEF by the 2021 updated ACC (American College of Cardiology) expert consensus (4) and the 2021 ESC (European Society of Cardiology) clinical guideline (5) because of their benefits in terms of improving cardiovascular outcomes demonstrated in many large-scale studies (6–9).

Network meta-analysis is an attractive statistical method that allows indirect comparison of multiple interventions that have not been investigated in a head-to-head manner. Recently published network meta-analyses have compared the efficacy of treatment regimens including ARNI, IVA, and conventional drugs in patients with HFrEF (10, 11). There have, however, been no studies comparing treatment regimens containing ARNI, IVA, and SGLT2i in patients with HFrEF.

This analysis used a network meta-analysis approach to compare the efficacy of treatment regimens including ARNI, IVA, SGLT2i, and conventional drugs in reducing HF hospitalization, cardiovascular mortality, and all-cause mortality in patients with chronic HFrEF.

## Methods

A systematic literature review was performed in accordance with the Preferred Reporting Items for Systematic Reviews and Meta-Analyses extension statement (PRISMA-NMA) (12).

### Study Selection and Identification

A literature search of PubMed, EMBASE, and the Cochrane Central Register of Controlled Trials (CENTRAL) from May 2017 to March 2021 was performed, using a search strategy adapted from the two reviews mentioned above (10, 11), which provided records from January 1987 to May 2017. Studies that included conventional and newer drugs for HFrEF, including ACEIs, BBs, ARBs, MRAs, ARNI, IVA, and SGLT2is (e.g., empagliflozin and dapagliflozin), were retrieved.

The included studies were randomized controlled trials (RCTs), conducted mainly in North America and Europe. The eligible population was limited to outpatients (aged ≥18 years) with chronic HFrEF [left ventricular ejection fraction (LVEF) ≤ 40%] of diverse etiology (e.g., ischemic and dilated cardiomyopathy). Studies were excluded if the entire study population had characteristics that would impact treatment response (e.g., hospitalized, acute HF, diabetes mellitus, coronary heart disease).

Data from the eligible studies were extracted by two reviewers and loaded into a database after reconciliation. The median or mean duration of the study, if reported, was preferentially extracted as the exposure duration; otherwise the prespecified follow-up time was used. For each outcome, the number of patients with at least one event during the follow-up period was extracted for each arm of the study. To avoid interference by concomitant drug classes of interest on treatment response, treatments were classified as including the concomitant drug when >50% of patients in the study were receiving the drug at baseline. In other words, if >50% of the trial patients received concomitant drugs of interest, the treatment was described as a combination therapy [study drug class(es) + concomitant drug class(es)] in the analysis.

### Network Meta-Analysis

Network meta-analysis, which includes direct and indirect study evidence, facilitates indirect comparisons of diverse interventions when direct evidence is lacking. For consistency, we used the same methodology as in the two previous network meta-analyses in HFrEF (10, 11), with a modeling framework proposed by Dias et al. (13) Data sets, including the mean or median follow-up time, the total number of patients randomized, and the numbers of patients with at least one event during the follow-up period for each arm, were entered into the model. By assuming an underlying Poisson process, the model used the log mean or median follow-up time to convert the probability of an event into a constant rate for each study arm and used a complementary clog-log (cloglog) link to model the event rates. Preference was given to presenting results from the random-effect model unless the fixed-effect model was more parsimonious than the random-effect model. Non-informative prior distributions were used. The analysis was conducted with published codes (13), using OpenBUGS version 3.2.3. Hazard ratios (HRs) and 95% credible intervals (95% CrIs) were presented after log inverse conversions of results from the Bayesian model. The probability that the treatment was better than the comparator (*P*-value) was determined after transformation of the 95% CrIs. Rank probabilities and expected rank were also presented.

## Results

### Study Search and Study Characteristics

**Figure 1:**
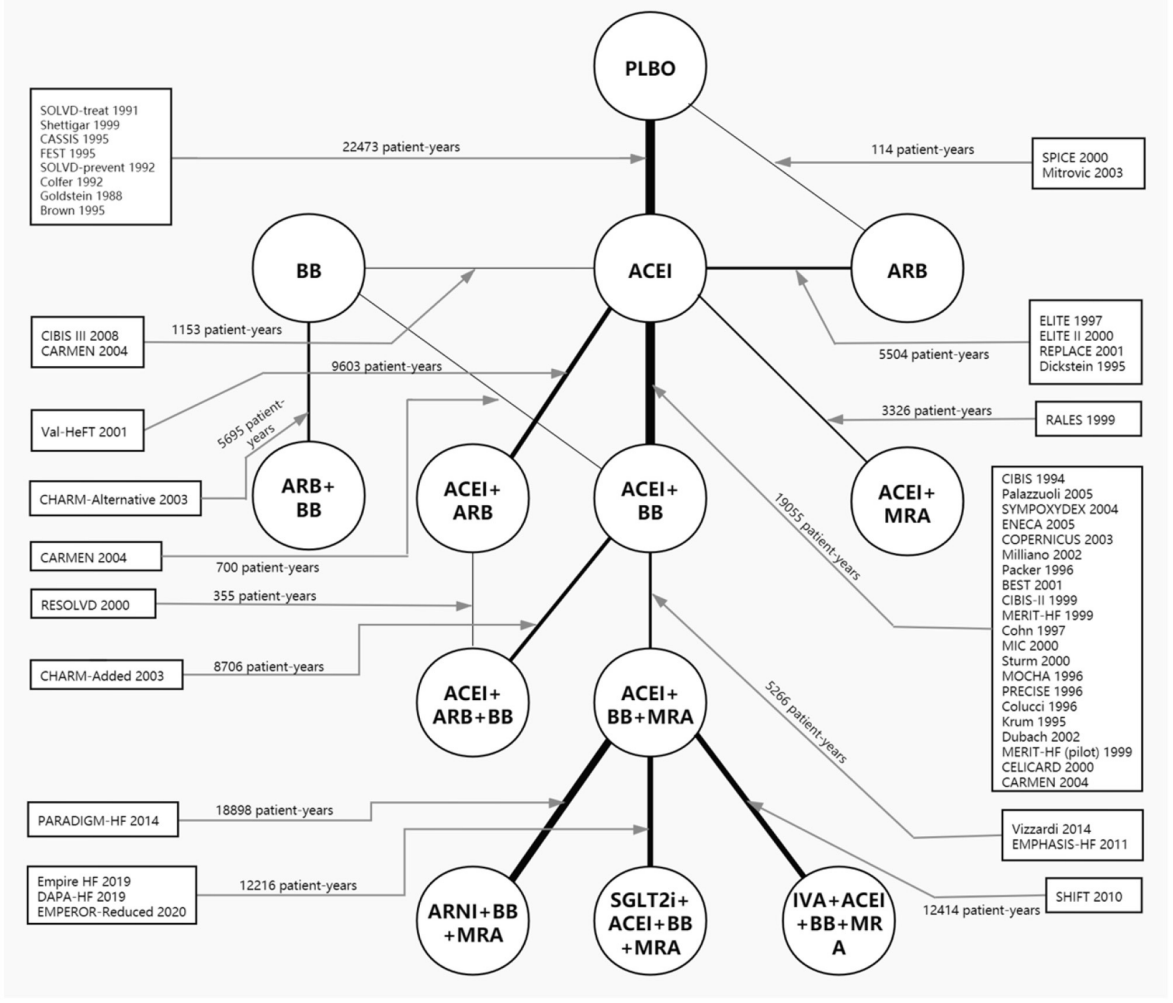
Network plot of all included studies reporting all-cause mortality. The thickness of the connecting lines was related to the number of patient-years of evidence for each intervention comparison. Network plots for cardiovascular mortality and hospital admission for heart failure are provided in Supplementary Figure 2. ACEI, angiotensin-converting enzyme inhibitor; ARB, angiotensin receptor blocker; ARNI, angiotensin receptor-neprilysin inhibitor; BB, beta-blocker; IVA, ivabradine; MRA, mineralocorticoid receptor antagonist; PLBO, placebo; SGLT2i, sodium-glucose cotransporter 2 inhibitor.

Forty-eight RCTs, comparing 13 treatments for HFrEF, were identified through retrieval and screening (Supplementary Figure 1), and a network diagram was constructed (Figure 1). Detailed information about the RCTs is listed in Supplementary Tables 1, 2. The eligible studies were mostly multicenter, double-blind, placebo-controlled trials performed in Europe and North America. A total of 68,074 patients were enrolled in the analysis, amounting to 125,477 patient-years. The sample size of the studies ranged from 28 to 8,399, with 18 studies including more than 1,000 participants and seven studies including <100 participants. The median follow-up time ranged from 2 to 44 months, with the median follow-up time of 13 trials being <6 months.

The baseline characteristics of the studies were stratified by interventions and are presented in Table 1. The age, sex, and LVEF of the populations of each intervention class were deemed similar. The HF severity distribution of each intervention class was mainly concentrated in moderate severity [New York Heart Association (NYHA) II/III]. Although the proportion of patients with NYHA II/III in the study on ACEI+MRA vs. ACEI was lower, the HF severity distribution was, overall, also deemed similar across the included intervention classes.

**Table 1:** Study population characteristics.

**Intervention class**	**No. of trials**	**Patients (randomized)**	**Follow-up (years)**	**Patient-years of evidence**	**Mean**			
					**Age (year)**	**Male (%)**	**NYHA II/III (%)**	**LVEF (%)**
ACEI vs. PLBO	8	8,176	2.7	22472.7	59.9	84.4	94.5	26.6
ARB vs. PLBO	2	488	0.2	113.8	60.5	72.2	97.2	27.5
ARB vs. ACEI	4	4,418	1.2	5504.3	70.8	70.9	95.7	30.2
BB vs. ACEI	2^*^	1,391	0.8	1153.0	69.7	71.8	97.8	28.8
ACEI+ARB vs. ACEI	1	5,010	1.9	9602.5	62.7	80.0	98.0	26.7
ACEI+BB vs. BB	1^*^	382	1.8	700.3	62.3	80.7	92.0	NA
ACEI+BB vs. ACEI	21^*^	15,681	1.2	19055.1	61.5	78.6	92.5	24.8
ACEI+MRA vs. ACEI	1	1,663	2.0	3326.0	65.0	73.2	70.0	25.4
ARB+BB vs. BB	1	2,028	2.8	5695.3	66.6	68.1	97.0	29.9
ACEI+ARB+BB vs. ACEI+ARB	1	426	0.8	355.0	61.5	82.2	92.0	28.5
ACEI+BB+MRA vs. ACEI+BB	2	2,867	1.8	5266.4	68.3	77.7	99.2	26.5
ACEI+ARB+BB vs. ACEI+BB	1	2,548	3.4	8705.7	64.0	78.7	97.0	28.0
ARNI+BB+MRA vs. ACEI+BB+MRA	1	8,399	2.3	18897.8	63.8	78.2	94.0	29.5
SGLT2i+ACEI+BB+MRA vs. ACEI+BB+MRA	3	8,664	1.4	12215.9	66.5	76.6	98.9	29.5
IVA+ACEI+BB+MRA vs. ACEI+BB+MRA	1	6,505	1.9	12413.7	60.4	76.4	99.0	29.0

*The baseline characteristics of population were stratified by the interventions and the comparators. ACEI, angiotensin-converting enzyme inhibitor; ARB, angiotensin receptor blocker; ARNI, angiotensin receptor-neprilysin inhibitor; BB, beta-blocker; IVA, ivabradine; LVEF, left ventricular ejection fraction; MRA, mineralocorticoid receptor antagonist; NYHA, New York Heart Association; PLBO, placebo; SGLT2i, sodium-glucose cotransporter 2 inhibitor*.

* *means that the intervention class includes a 3-arm trial*.

### Network Meta-Analysis

**Figure 2:**
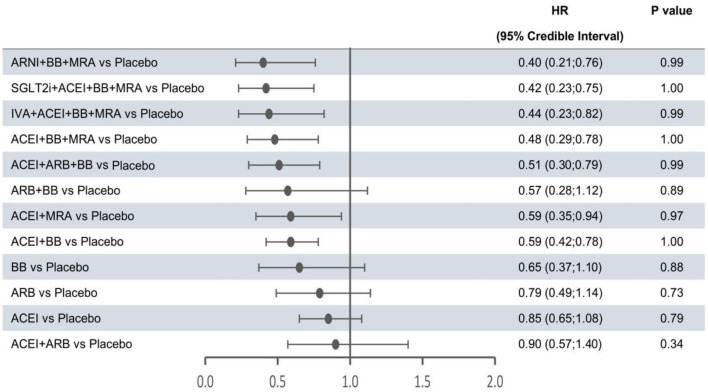
Efficacy of treatments vs. placebo in all-cause mortality. All-cause mortality was reported as hazard ratios (HRs) and 95% credible intervals for treatment vs. placebo. *P*-values were calculated based on the 95% credible intervals of hazard ratios and indicated the probability that the treatment was better than placebo. ACEI, angiotensin-converting enzyme inhibitor; ARB, angiotensin receptor blocker; ARNI, angiotensin receptor-neprilysin inhibitor; BB, beta-blocker; IVA, ivabradine; MRA, mineralocorticoid receptor antagonist; SGLT2i, sodium-glucose cotransporter 2 inhibitor.

**Figure 3:**
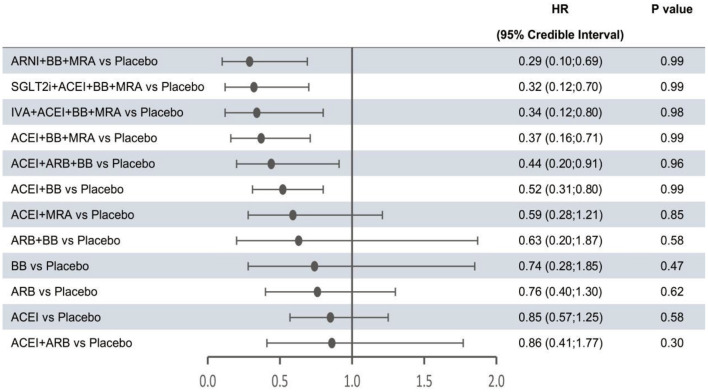
Efficacy of interventions vs. placebo in cardiovascular mortality. Cardiovascular mortality was reported as hazard ratios (HRs) and 95% credible intervals for treatment vs. placebo. *P*-values were calculated based on the 95% credible intervals of hazard ratios and indicated the probability that the treatment was better than placebo. ACEI, angiotensin-converting enzyme inhibitor; ARB, angiotensin receptor blocker; ARNI, angiotensin receptor-neprilysin inhibitor; BB, beta-blocker; IVA, ivabradine; MRA, mineralocorticoid receptor antagonist; SGLT2i, sodium-glucose cotransporter 2 inhibitor.

**Figure 4:**
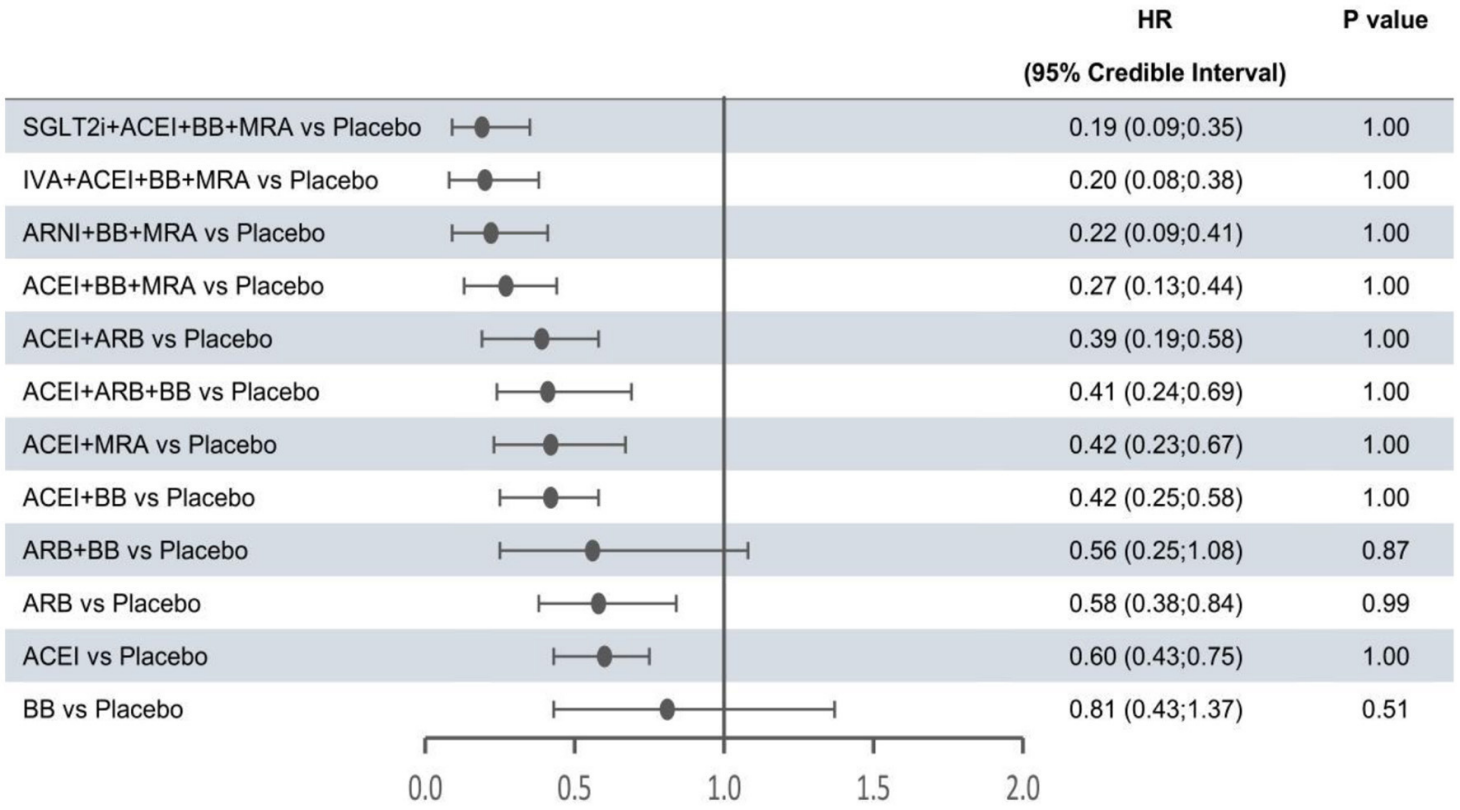
Efficacy of interventions vs. placebo in hospital admission for heart failure. Hospital admission for heart failure was reported as hazard ratios (HRs) and 95% credible intervals for treatment vs. placebo. *P*-values were calculated based on the 95% credible intervals of hazard ratios and indicated the probability that the treatment was better than placebo. ACEI, angiotensin-converting enzyme inhibitor; ARB, angiotensin receptor blocker; ARNI, angiotensin receptor-neprilysin inhibitor; BB, beta-blocker; IVA, ivabradine; MRA, mineralocorticoid receptor antagonist; SGLT2i, sodium-glucose cotransporter 2 inhibitor.

In the network meta-analysis, all results for each outcome were estimated based on direct and indirect evidence. In the network of evidence, between-study heterogeneity for each outcome was found to reach statistical significance (namely, the 95% CrIs of heterogeneity do not contain 0), which was expected given the differences in inclusion criteria, design and endpoint adjudication across the included studies. Still, the size of heterogeneity was considered low and acceptable. The heterogeneity parameters (SD) for all-cause mortality, cardiovascular mortality, and hospitalization for HF were 0.17 (95% CrI 0.05–0.35), 0.26 (95% CrI 0.07–0.53), and 0.16 (95% CrI 0.01–0.47), respectively. Comparative efficacies of 12 intervention classes vs. placebo in terms of these three outcomes are shown in Figures 2–4 and complete results for the three outcomes are presented in Supplementary Table 3.

The forest plots shown in Figures 2–4 were drawn on the basis of the distance from the point estimate of each intervention to the null-effect line (1.0), with the intervention at the top being the most effective. The results for all-cause mortality (Figure 2) demonstrated that combination treatments, except for ACEI+ARB and ARB+BB, were significantly superior to placebo, based on HRs and 95% CrIs. Considering the *P*-values, each intervention, except for ACEI+ARB, was quite likely to be better than placebo, in agreement with the earlier perspective. Of the 12 interventions, ARNI+BB+MRA, SGLT2i+ACEI+BB+MRA, and IVA+ACEI+BB+MRA were the best therapies, with risk reductions of 60, 58, and 56%, respectively, in all-cause mortality, compared with placebo. As shown in Figure 3, the trend of point estimates for cardiovascular mortality was similar to that for all-cause mortality. Nevertheless, the probability that monotherapies were better than placebo for cardiovascular mortality was lower than that for all-cause mortality. The differences in efficacy of interventions in reducing hospitalization for HF (Figure 4) were more significant than differences in efficacy in reducing all-cause or cardiovascular mortality. The best combinations for reducing hospitalization for HF were SGLT2i+ACEI+BB+MRA, IVA+ACEI+BB+MRA, and ARNI+BB+MRA, with reductions of 81, 80, and 78%, respectively. Fewer studies reported cardiovascular mortality (33 RCTs) or HF hospitalizations (27 RCTs) than all-cause mortality (48 RCTs). The results for all-cause mortality were, therefore, probably a little more reliable than those for the other two outcomes.

**Figure 5:**
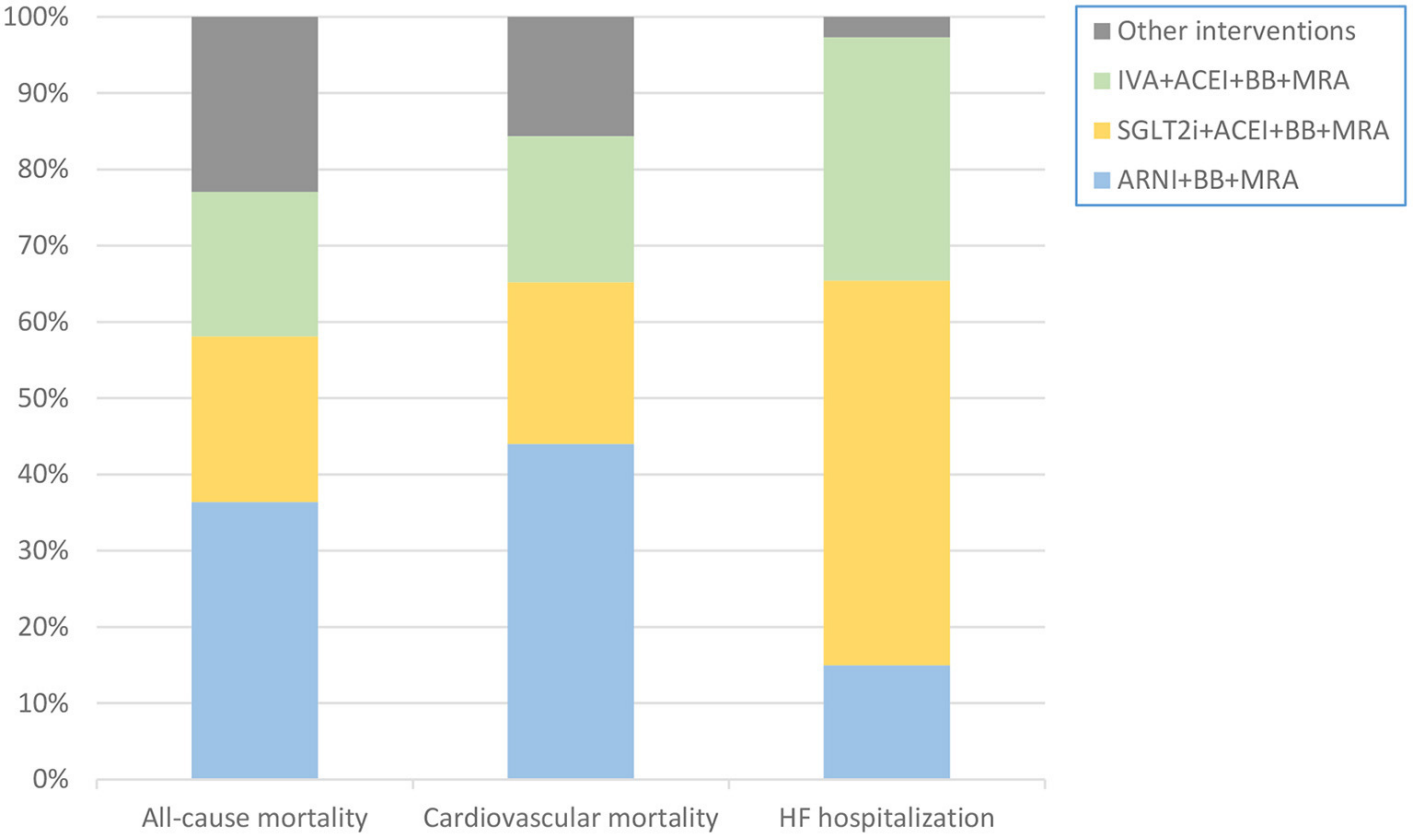
Stacking histogram showing probability that each intervention is the best therapy in each outcome. The probability that ARNI+BB+MRA was the optimal treatment was highest in terms of reducing all-cause or cardiovascular mortality. The probability that SGLT2i+ACEI+BB+MRA was the optimal treatment was highest in terms of reducing hospitalization for HF. ACEI, angiotensin-converting enzyme inhibitor; ARNI, angiotensin receptor-neprilysin inhibitor; BB, beta-blocker; HF, heart failure; IVA, ivabradine; MRA, mineralocorticoid receptor antagonist; SGLT2i, sodium-glucose cotransporter 2 inhibitor.

The probabilities that a particular class of intervention was the optimal treatment are presented in Figure 5. ARNI+BB+MRA had the highest probability of being the optimal treatment in terms of reducing the risk of death from any cause or cardiovascular causes, and SGLT2i+ACEI+BB+MRA had the highest probability of being the optimal treatment in terms of reducing hospitalization for HF. The probability graphs (Supplementary Figure 3), showing rank probabilities and mean rank for each treatment, were consistent with this conclusion.

## Discussion

The emergence of increasing numbers of new classes of drugs to treat HFrEF makes it difficult for cardiologists to determine the optimal medication regimen for patients with HFrEF. To assist clinicians in selecting the optimum regimen, we have performed a network meta-analysis to evaluate the efficacy of 13 classes of interventional drugs on hospitalization for HF, cardiovascular mortality, and all-cause mortality. Two earlier network analyses evaluated the efficacy of 12 intervention classes other than SGLT2i+ACEI+BB+MRA in patients with HF and LVEF ≤ 45%. According to current guidelines, however, experts tend to define HFrEF as HF with LVEF ≤ 40% (4, 5), which meant that, in a strict sense, the patients enrolled in these earlier studies included a fraction of patients with HF and mid-range ejection fraction (HFmrEF). Although patients with HFmrEF share many features with patients with HFrEF, the outcomes of patients with HFmrEF are more similar to patients with HF and preserved ejection fraction (5). In the present analysis, the study population was limited to patients with LVEF ≤ 40%, and studies on SGLT2is were added. This is the first study to indirectly compare the efficacy of therapeutic regimens including SGLT2is with the efficacy of therapeutic regimens including ARNI, IVA, and conventional drugs, using a network meta-analysis method.

This analysis focused on providing data on the estimated efficacy of combined therapies or monotherapies of SGLT2i, ARNI, IVA, and conventional drugs in patients with HFrEF, compared with placebo. Three combined therapies (SGLT2i+ACEI+BB+MRA, IVA+ACEI+BB+MRA, and ARNI+BB+MRA) were found to be the best therapies for each outcome in patients with HFrEF. Overall, the benefits of the three combined treatments in improving cardiovascular outcomes were similar, although there were small differences in their efficacy in individual outcomes. ARNI+BB+MRA probably had more advantages in decreasing mortality, whereas SGLT2i+ACEI+BB+MRA might be superior to other intervention classes in reducing hospitalization for HF.

Because of their similar efficacies, one of these three therapeutic regimens should be selected based mainly on whether any of the included drugs is incompatible with the background therapy of the patient. It is worth noting that ARNI and ACEI could not be combined in patients with HFrEF because the combination of these two drug classes was likely to lead to a significantly increased incidence of angioedema. ACEI treatment must be stopped for 36 h before initiation of ARNI (4, 5), leaving a window of uncertainty in terms of patient safety. Under circumstances where the indications for all three new interventions are met, the combinations containing SGLT2i or IVA may be more suitable for patients with HFrEF who have been using ACEI but still have clinical symptoms.

Although SGLT2is were initially developed as antidiabetic drugs, increasing numbers of clinical trials have demonstrated additional benefits, including cardiovascular and renal benefits (8, 14–20). In one large-scale study, approximately 40% of individuals with chronic HF had ≥5 non-cardiovascular comorbidities (e.g., renal disease and diabetes), which probably interacted with HF and thus resulted in more adverse outcomes (21). The emergence of SGLT2is, to some degree, solves this problem. The 2021 guidelines have recommended SGLT2is for patients with HFrEF and renal disease or type 2 diabetes because of their unique superiority in this subpopulation (4, 5).

According to the guideline, SGLT2i+ARNI+BB+MRA is deemed to be the optimal combination if the indications for these drugs are all met (4, 5). A cross-trial analysis that indirectly compared this combination with ACEI/ARB+BB demonstrated that SGLT2i+ARNI+BB+MRA significantly improved cardiovascular outcomes in HFrEF patients (22). There is, however, no direct study evidence showing that SGLT2i+ARNI+BB+MRA provides better efficacy than other combined therapies. Based on our results, it can be speculated that SGLT2i+ARNI+BB+MRA is the optimal therapy. SGLT2is and the neprilysin inhibitor component of ARNI shared many beneficial mechanisms (e.g., natriuresis, increased lipolysis, and anti-inflammatory activity) (23, 24), which may mean that there is less benefit in combining an SGLT2i with ARNI. Although the efficacy of ARNI was better than that of ACEI, it was uncertain whether the efficacy of SGLT2i+ARNI+BB+MRA was better than that of SGLT2i+ACEI+BB+MRA. In our analysis, the efficacy of ARNI+BB+MRA was not generally lower than that of SGLT2i+ACEI+BB+MRA, most notably in reducing mortality. Although the benefit of combining an SGLT2i and ARNI is likely to be smaller, the combined therapy of SGLT2i and ARNI contributed to more cardiovascular benefits than ARNI monotherapy (25). It is, therefore, highly probable that SGLT2i+ARNI+BB+MRA is better than SGLT2i+ACEI+BB+MRA.

The use of multiple drugs in patients with HFrEF may contribute to hypotension. In the PARADIGM-HF (9) (Prospective Comparison of ARNI with ACEI to Determine Impact on Global Mortality and Morbidity in Heart Failure) trial, ARNI led to a significant increase in the risk of hypotension or symptomatic hypotension, compared with ACEI, when added to BB+MRA. However, in the EMPEROR-Reduced (6) (Empagliflozin Outcome Trial in Patients with Chronic Heart Failure and a Reduced Ejection Fraction) trial, SGLT2i+ACEI+BB+MRA showed similar risks to ACEI+BB+MRA in terms of hypotension or symptomatic hypotension. Therefore, the SGLT2i+ACEI+BB+MRA may be a better choice for patients who cannot tolerate ARNI because of hypotension.

## Limitations

In this analysis, 13 studies with a follow-up <6 months and seven studies enrolling <100 patients were included and the limitation on eligible population meant that a few high-quality studies were excluded, which probably led to an imprecise estimation of efficacy for a fraction of the interventions. A study on ACEI+MRA vs. ACEI included fewer patients with NYHA II/III and more patients with NYHA IV, probably resulting in underestimation of efficacy of ACEI+MRA but little effect on the whole. Differences between different drugs in the same class were disregarded, as were differences in doses of the drugs. Since HF patients coming from RCT are different from HF patients in real world, the results of our study are for reference only.

The combined endpoint of cardiovascular death or hospitalization for HF has been used as the primary outcome in many recent large-scale trials (6–9) because this outcome can comprehensively demonstrate the efficacy of an intervention. Because of the scarcity of studies reporting this outcome, we were unable to evaluate the results for this outcome. Moreover, none of the eligible studies with SGLT2is reported the number of patients hospitalized for any reason and, although this outcome was reported in the previous study, the results for the outcome were not shown in the present analysis.

Because current use of the new drug ARNI is not universal, no study on SGLT2i+ARNI+BB+MRA was included and this network meta-analysis could not directly compare this combination with the 13 included interventions and thus evaluate the efficacy of this combination.

The start time of literature retrieval was not database inception, which probably led to omission of a fraction of relevant studies. The included studies covered a time span of approximately 35 years, during which time many uncontrollable factors (e.g., environment and diet) have changed, potentially affecting population characteristics.

## Conclusions

Our analysis indicated that three combined treatments (SGLT2i+ACEI+BB+MRA, IVA+ACEI+BB+MRA, and ARNI+BB+MRA) were the most effective among 13 intervention classes in reducing mortality and HF hospitalization for HFrEF patients. Although the efficacies of the three combined therapies were overall similar, ARNI+BB+MRA was more likely to be the optimal therapy in reducing mortality, and SGLT2i+ACEI+BB+MRA was more likely to be the optimal therapy in decreasing hospitalization for HF. Our results of this analysis were in line with the latest guideline recommendation. The increasing use of combinations of conventional and novel drugs contributed to progressive reductions in hospitalization and mortality in patients with HFrEF.

## Data Availability Statement

The original contributions presented in the study are included in the article/Supplementary Materials, further inquiries can be directed to the corresponding author/s.

## Author Contributions

BX wrote the manuscript. BX and ZY performed the statistical analysis. XZ revised the manuscript. All authors contributed to the article and approved the submitted version.

## Funding

This work was funded by the Talent Support Project of the Second Affiliated Hospital of Soochow University (XKTJ-RC202003).

## Conflict of Interest

The authors declare that the research was conducted in the absence of any commercial or financial relationships that could be construed as a potential conflict of interest.

## Publisher's Note

All claims expressed in this article are solely those of the authors and do not necessarily represent those of their affiliated organizations, or those of the publisher, the editors and the reviewers. Any product that may be evaluated in this article, or claim that may be made by its manufacturer, is not guaranteed or endorsed by the publisher.
