# Supplementary material for: Comparative Efficacy of Medical Treatments for Chronic Heart Failure: A Network Meta-Analysis
Source: Front Cardiovasc Med. 2022 Jan 13;8:787810. doi: 10.3389/fcvm.2021.787810 (PMC8793336; doi:10.3389/fcvm.2021.787810)
Supplement: Supplementary file 1 [file Data_Sheet_1.docx]

**Figure S1.** **Flow diagram of study retrieval and identification for network meta-analysis.**

**
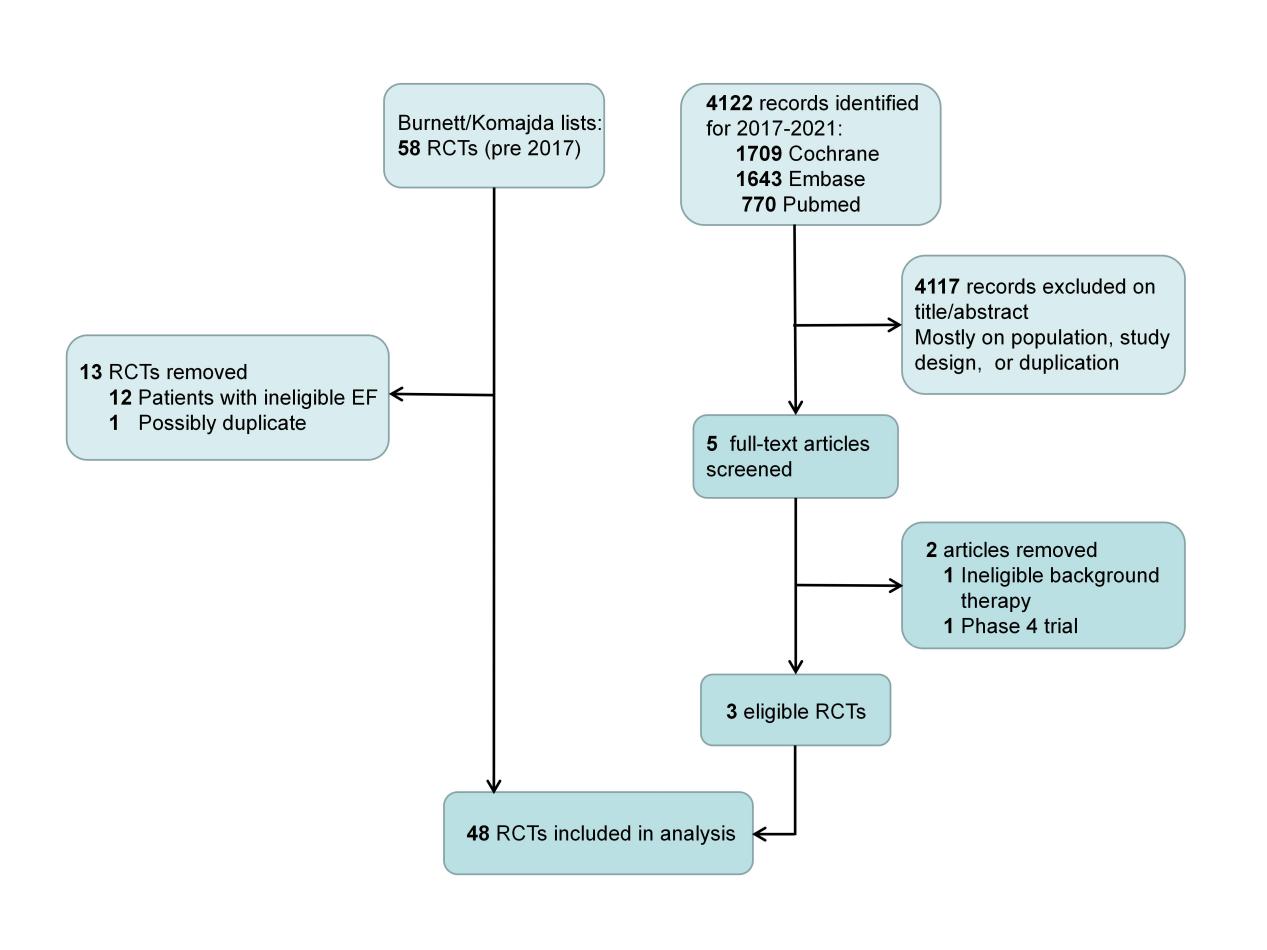
**

**Figure S2. Network plots of the included studies reporting cardiovascular mortality (A) and hospitalization for heart failure (B)**

**
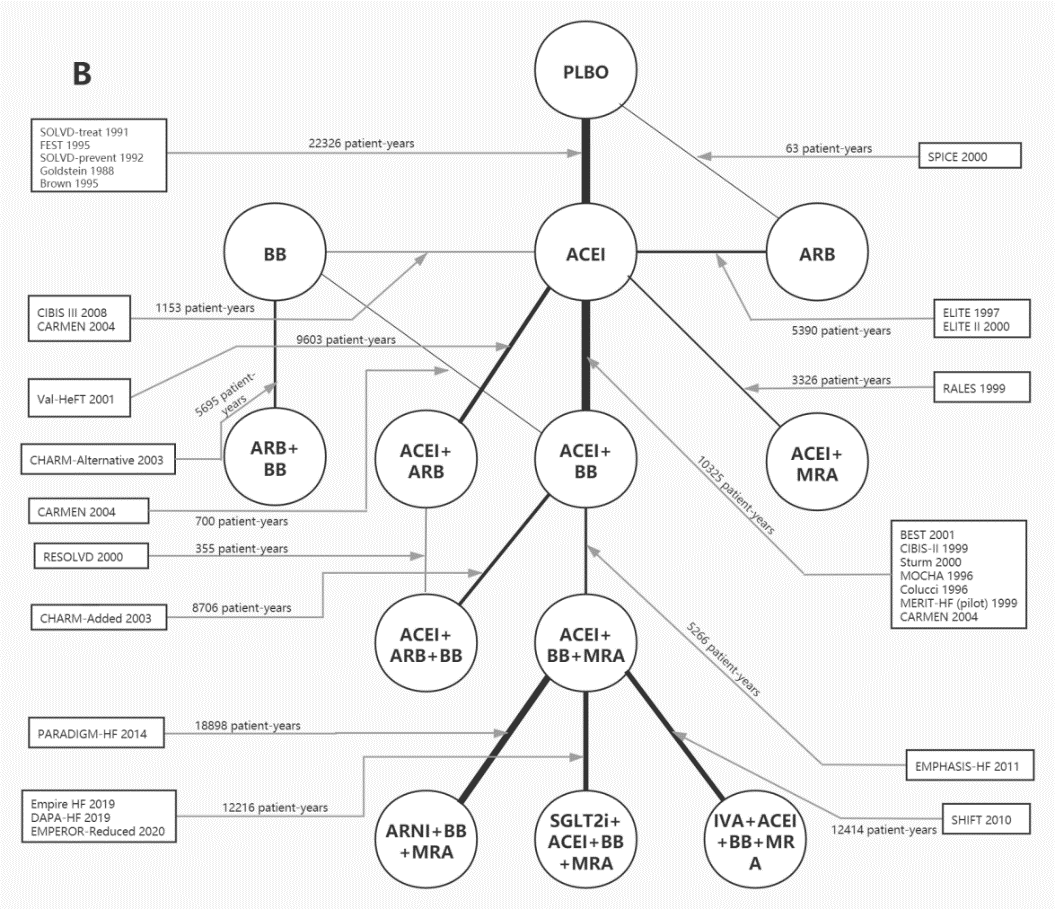

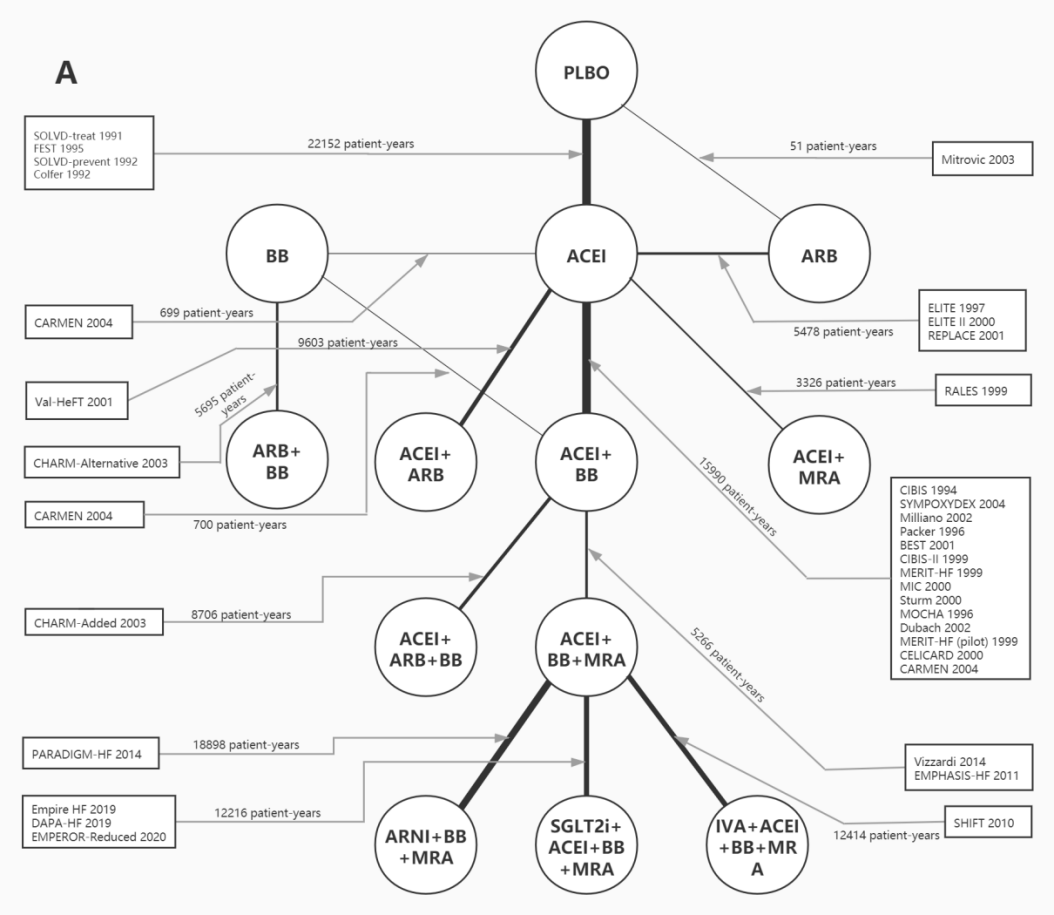
**

**Figure S3. Results of network meta-analysis probability rank for all-cause mortality (A), cardiovascular mortality (B), and hospitalization for heart failure (C).**

**Table S1. Study design and baseline population characteristics of included RCTs.**

| **Trial/Author year** | **Study design** | **# centers/ location** | **Median follow-up (m)** | **Number** | **Treatment** | **Daily dosage** | **Age**  **(y)** | **Men (%)** | **NYHA II/III (%)** | **Ejection fraction (%)** | **LVEF inclusion criteria** | **eGFR (ml/min/**  **1.73 m^2)^** | **Ischemic HF (%)** | **Prior MI (%)** | **HF duration**  **(m)** | **DM (%)** |
| --- | --- | --- | --- | --- | --- | --- | --- | --- | --- | --- | --- | --- | --- | --- | --- | --- |
| **DAPA-HF 2019^1^** | QB, MC, PC | 410/ 20 countries | 18.2 | 2373 | Dapagliflozin | 10mg | 66.3 | 76.6 | 99.0 | 31.1 | ≤ 40% | 65.8 | 56.4 | NA | NA | 41.8 |
|  |  |  |  | 2371 | Placebo | NA |  |  |  |  |  |  |  |  |  |  |
| **EMPEROR-Reduced 2020^2^** | DB, MC, PC | 520/ 20 countries | 16.0 | 1863 | Empagliflozin | 10mg | 66.8 | 76.1 | 99.0 | 27.4 | ≤ 40% | 62.0 | 51.7 | NA | NA | 49.8 |
|  |  |  |  | 1867 | Placebo | NA |  |  |  |  |  |  |  |  |  |  |
| **Empire HF 2019^3^** | DB, MC, PC | 2/ Denmark | 3.0 | 95 | Empagliflozin | 10mg | 63.5 | 85.3 | 94.0 | 30.0 | ≤ 40% | 73.5 | 51.1 | NA | 31 | 17.4 |
|  |  |  |  | 95 | Placebo | NA |  |  |  |  |  |  |  |  |  |  |
| **PARADIGM-HF 2014^4^** | DB, MC | 1043/ 46 countries | 27.0 | 4187 | LCZ696 | 400mg | 63.8 | 78.2 | 94.0 | 29.5 | ≤ 40% | NA | 60.0 | 43.2 | NA | 34.6 |
|  |  |  |  | 4212 | Enalapril | 20mg |  |  |  |  |  |  |  |  |  |  |
| **RALES 1999^5^** | DB, MC, PC | 195/ 15 countries | 24.0 | 822 | Spironolactone | 25-50mg | 65.0 | 73.2 | 70.0 | 25.4 | ≤ 35% | NA | 54.5 | NA | NA | NA |
|  |  |  |  | 841 | Placebo | NA |  |  |  |  |  |  |  |  |  |  |
| **Vizzardi 2014^6^** | SB, SC, PC | 1/ Italy | 44.0 | 65 | Spironolactone | 25-400mg | 62.2 | NA | 82.0 | 36.0 | < 40% | 77.1 | NA | NA | NA | 13.8 |
|  |  |  |  | 65 | Placebo | 25mg |  |  |  |  |  |  |  |  |  |  |
| **EMPHASIS-HF 2011^7^** | DB, MC, PC | 278/ 29 countries | 21.0 | 1364 | Eplerenone | 50mg | 68.6 | 77.7 | 100.0 | 26.1 | ≤ 35% | 70.8 | 68.9 | 50.5 | NA | 31.4 |
|  |  |  |  | 1373 | Placebo | NA |  |  |  |  |  |  |  |  |  |  |
| **Val-HeFT 2001^8^** | DB, MC, PC | 302/ 16 countries | 23.0 | 2511 | Valsartan | 320mg | 62.7 | 80.0 | 98.0 | 26.7 | < 40% | NA | 57.2 | 57.2 | NA | 25.5 |
|  |  |  |  | 2499 | Placebo | NA |  |  |  |  |  |  |  |  |  |  |
| **SPICE 2000^9^** | SB, MC, PC | 90/ 7 countries | 2.8 | 179 | Candesartan | 16mg | 65.7 | 68.9 | 95.0 | 27.0 | < 35% | NA | 71.5 | 62.2 | NA | 18.9 |
|  |  |  |  | 91 | Placebo | NA |  |  |  |  |  |  |  |  |  |  |
| **CHARM-Alternative 2003^10^** | DB, MC, PC | 618/ 26 countries | 33.7 | 1013 | Candesartan | 32mg | 66.6 | 68.1 | 97.0 | 29.9 | ≤ 40% | NA | 68.3 | 61.5 | NA | 27.0 |
|  |  |  |  | 1015 | Placebo | NA |  |  |  |  |  |  |  |  |  |  |
| **Mitrovic 2003^11^** | DB, MC | NA/Europe | 2.8 | 174 | Candesartan | 2-16mg | 54.0 | 85.3 | 100.0 | 28.1 | ≤ 40% | NA | NA | NA | 40 | NA |
|  |  |  |  | 44 | Placebo | NA |  |  |  |  |  |  |  |  |  |  |
| **CHARM-Added 2003^12^** | DB, MC, PC | 618/ 26 countries | 41.0 | 1276 | Candesartan | 32mg | 64.0 | 78.7 | 97.0 | 28.0 | ≤ 40% | NA | 62.4 | 55.6 | NA | 29.7 |
|  |  |  |  | 1272 | Placebo | NA |  |  |  |  |  |  |  |  |  |  |
| **SOLVD-treat 1991^13^** | DB, MC, PC | 23/ US, Canada, Belgium | 41.4 | 1285 | Enalapril | 20mg | 60.8 | 80.4 | 87.0 | 24.8 | ≤ 35% | NA | 71.1 | 65.7 | NA | 25.8 |
|  |  |  |  | 1284 | Placebo | NA |  |  |  |  |  |  |  |  |  |  |
| **Shettigar 1999^14^** | DB, MC, PC | 28/ US | 2.8 | 102 | Fosinopril | 40mg | 62.0 | 75.0 | 91.0 | 24.0 | ≤ 35% | NA | 43.0 | 34.0 | NA | NA |
|  |  |  |  | 104 | Placebo | NA |  |  |  |  |  |  |  |  |  |  |
| **CASSIS 1995^15^** | DB, MC, PC | 18/ Czech and  Slovak Rep | 2.8 | 200 | Spirapril, Enalapril | E:10mg S:1.5-6mg | 57.5 | 83.0 | 81.0 | 28.0 | ≤ 40% | NA | 70.0 | 46.0 | NA | 23.0 |
|  |  |  |  | 48 | Placebo | NA |  |  |  |  |  |  |  |  |  |  |
| **FEST 1995^16^** | DB, MC, PC | 42/ 8 countries | 2.8 | 155 | Fosinopril | 40mg | 63.5 | 74.4 | 100.0 | 26.5 | ≤ 35% | NA | NA | NA | NA | NA |
|  |  |  |  | 153 | Placebo | NA |  |  |  |  |  |  |  |  |  |  |
| **SOLVD-prevent 1992^17^** | DB, MC, PC | 23/ US, Canada, Belgium | 37.4 | 2111 | Enalapril | 20mg | 59.1 | 88.6 | 100.0 | 28.0 | ≤ 35% | NA | 83.2 | 79.9 | NA | 15.2 |
|  |  |  |  | 2117 | Placebo | NA |  |  |  |  |  |  |  |  |  |  |
| **Brown 1995^18^** | DB, MC, PC | 41/ US | 5.6 | 116 | Fosinopril | 20mg | 62.0 | 79.7 | 91.0 | 25.0 | ≤ 35% | NA | NA | NA | NA | NA |
|  |  |  |  | 125 | Placebo | NA |  |  |  |  |  |  |  |  |  |  |
| **Colfer 1992^19^** | DB, MC, PC | 22/ US | 2.8 | 114 | Benazepril | 20mg | 61.7 | 83.1 | 89.0 | 25.0 | ≤ 35% | NA | 55.8 | NA | 48 | NA |
|  |  |  |  | 58 | Placebo | NA |  |  |  |  |  |  |  |  |  |  |
| **Goldstein 1988^20^** | DB, SC, PC | NA/ NA | 6.0 | 104 | Captopril | 150mg | 56.0 | 81.6 | 96.0 | 24.8 | ≤ 40% | NA | 63.0 | NA | 35 | NA |
|  |  |  |  | 100 | Placebo | NA |  |  |  |  |  |  |  |  |  |  |
| **ELITE 1997^21^** | DB, MC | 125/ US, Europe, South America | 11.0 | 352 | Losartan | 50mg | 73.5 | 66.8 | 99.0 | 30.5 | ≤ 40% | NA | 68.1 | 50.0 | NA | 25.3 |
|  |  |  |  | 370 | Captopril | 150mg |  |  |  |  |  |  |  |  |  |  |
| **ELITE** **II 2000^22^** | DB, MC | 289/ 46 countries | 18.0 | 1578 | Losartan | 50mg | 71.4 | 69.3 | 95.0 | 31.0 | ≤ 40% | NA | 79.0 | 58.4 | NA | 23.8 |
|  |  |  |  | 1574 | Captopril | 150mg |  |  |  |  |  |  |  |  |  |  |
| **REPLACE 2001^23^** | DB, MC | NA/ Europe, Israel | 2.8 | 301 | Telmisartan | 10-80mg | 64.0 | 89.0 | 100.0 | 26.4 | ≤ 40% | NA | NA | NA | NA | NA |
|  |  |  |  | 77 | Enalapril | 20mg |  |  |  |  |  |  |  |  |  |  |
| **Dickstein 1995^24^** | DB, MC | 19/ Denmark, Finland, Norway, Sweden | 1.9 | 108 | Losartan | 25-50mg | 64.3 | 77.5 | 84.0 | 23.3 | ≤ 35% |  | 69.4 | 62.6 | 46 | NA |
|  |  |  |  | 58 | Enalapril | 20mg |  |  |  |  |  |  |  |  |  |  |
| **CIBIS 1994^25^** | DB, MC, PC | NA/ Europe | 22.8 | 320 | Bisoprolol | 5mg | 59.6 | 82.5 | 95.0 | 25.4 | ≤ 40% | NA | 54.6 | 47.3 | 38 | NA |
|  |  |  |  | 321 | Placebo | NA |  |  |  |  |  |  |  |  |  |  |
| **RESOLVD 2000^26^** | DB, MC, PC | 60/ North America, Italy | 10.0 | 214 | Metoprolol+(  candesartan, enalapril, or candesartan+  enalapril) | 200mg | 61.5 | 82.2 | 92.0 | 28.5 | ≤ 40% | NA | 69.2 | 63.6 | NA | 25.4 |
|  |  |  |  | 212 | Placebo+(  candesartan, enalapril, or candesartan+  enalapril) | NA |  |  |  |  |  |  |  |  |  |  |
| **Palazzuoli 2005^27^** | DB, PC | NA/ Italy | 12.0 | 32 | Carvedilol | 50mg | 71.0 | 65.5 | 57.0 | 32.0 | < 40% | NA | 69.0 | NA | NA | NA |
|  |  |  |  | 26 | Placebo | NA |  |  |  |  |  |  |  |  |  |  |
| **SYMPOXYDEX 2004^28^** | DB, MC, PC | NA/ France | 6.0 | 28 | Carvedilol | 50mg | 59.4 | 84.0 | 100.0 | 25.9 | < 40% | NA | 40.0 | NA | NA | NA |
|  |  |  |  | 22 | Placebo | NA |  |  |  |  |  |  |  |  |  |  |
| **ENECA 2005^29^** | DB, MC, PC | 70/ NA | 8.0 | 134 | Nebivolol | 10mg | 72.1 | 73.5 | 96.0 | 25.9 | < 35% | NA | NA | 58.5 | NA | 25.8 |
|  |  |  |  | 126 | Placebo | NA |  |  |  |  |  |  |  |  |  |  |
| **COPERNICUS 2003^30^** | DB, MC, PC | 334/ North America, Europe, Australia | 12.0 | 1156 | Carvedilol | 50mg | 63.3 | 79.7 | NA | 20.0 | < 20% | NA | 67.2 | NA | NA | NA |
|  |  |  |  | 1133 | Placebo | NA |  |  |  |  |  |  |  |  |  |  |
| **Dubach 2002^31^** | DB, PC | NA | 12.0 | 13 | Bisoprolol | 10mg | 57.5 | NA | 100.0 | 25.8 | < 40% | NA | 57.1 | NA | NA | 3.6 |
|  |  |  |  | 15 | Placebo | NA |  |  |  |  |  |  |  |  |  |  |
| **Milliano 2002^32^** | DB, MC, PC | NA/ Netherlands | 6.0 | 43 | Metoprolol | 50-150mg | 64.8 | 66.6 | 100.0 | 25.2 | < 35% | NA | 55.5 | NA | NA | NA |
|  |  |  |  | 11 | Placebo | NA |  |  |  |  |  |  |  |  |  |  |
| **Packer 1996^33^** | DB, MC, PC | NA/ US | 6.5 | 696 | Carvedilol | 50-100mg | 58.0 | 76.6 | 97.0 | 22.6 | ≤ 35% | NA | 47.6 | NA | NA | NA |
|  |  |  |  | 398 | Placebo | NA |  |  |  |  |  |  |  |  |  |  |
| **BEST 2001^34^** | DB, MC, PC | 90/ US, Canada | 24.0 (mean) | 1354 | Bucindolol | 100mg or 200mg | 60.0 | 78.1 | 92.0 | 23.0 | ≤ 35% | NA | 58.6 | NA | 37 | 35.6 |
|  |  |  |  | 1354 | Placebo | NA |  |  |  |  |  |  |  |  |  |  |
| **MERIT-HF (pilot) 1999^35^** | DB, MC, PC | NA | 6.0 | 42 | Metoprolol | 150mg | NA | 75.4 | 97.0 | 27.0 | < 40% | NA | 36.1 | NA | NA | NA |
|  |  |  |  | 19 | Placebo | NA |  |  |  |  |  |  |  |  |  |  |
| **CELICARD 2000^36^** | DB, MC, PC | NA/ Poland, France | 12.0 | 62 | Celiprolol | 100mg | 56.5 | 89.5 | 100.0 | 26.0 | < 40% | NA | NA | 40.0 | NA | NA |
|  |  |  |  | 62 | Placebo | NA |  |  |  |  |  |  |  |  |  |  |
| **CIBIS-II 1999^37^** | DB, MC, PC | 47/ Europe | 15.6 | 1327 | Bisoprolol | 2.5-10mg | 61.0 | 80.5 | 83.0 | 27.5 | ≤ 35% | NA | 49.7 | NA | 43 | NA |
|  |  |  |  | 1320 | Placebo | NA |  |  |  |  |  |  |  |  |  |  |
| **MERIT-HF 1999^38^** | DB, MC, PC | 313/ Europe, US | 12.0 | 1990 | Metoprolol | 200mg | 63.8 | 77.5 | 96.0 | 28.0 | ≤ 40% | NA | 62.3 | 48.2 | NA | 24.7 |
|  |  |  |  | 2001 | Placebo | NA |  |  |  |  |  |  |  |  |  |  |
| **Cohn 1997^39^** | DB, MC, PC | 42/ US | 3.0 | 70 | Carvedilol | 50mg | 60.0 | 58.1 | 87.0 | 22.0 | ≤ 35% | NA | 44.8 | NA | 49 | NA |
|  |  |  |  | 35 | Placebo | NA |  |  |  |  |  |  |  |  |  |  |
| **MIC 2000^40^** | DB, MC, PC | NA/ Germany, Sweden | 6.0 | 26 | Metoprolol | 135mg(mean) | 54.0 | 71.2 | 94.0 | 27.9 | < 40% | NA | NA | NA | NA | NA |
|  |  |  |  | 26 | Placebo | NA |  |  |  |  |  |  |  |  |  |  |
| **Sturm 2000^41^** | DB, SC, PC | 1/ Austria | 24.0 | 51 | Atenolol | 50-100mg | 51.5 | 88.0 | 98.0 | 17.0 | ≤ 25% | NA | 28.0 | NA | NA | 18.0 |
|  |  |  |  | 49 | Placebo | NA |  |  |  |  |  |  |  |  |  |  |
| **MOCHA 1996^42^** | DB, MC, PC | NR/ US | 6.0 | 261 | Carvedilol | 12.5-50mg | 59.5 | 76.0 | 98.0 | 23.0 | ≤ 35% | NA | 52.1 | NA | 57 | NA |
|  |  |  |  | 84 | Placebo | NA |  |  |  |  |  |  |  |  |  |  |
| **PRECISE 1996^43^** | DB, MC, PC | 31/ US | 6.0 | 145 | Carvedilol | 50mg | 60.3 | 73.4 | 96.0 | 22.0 | ≤ 35% | NA | 51.8 | NA | NA | NA |
|  |  |  |  | 133 | Placebo | NA |  |  |  |  |  |  |  |  |  |  |
| **Colucci 1996^44^** | DB, MC, PC | NA/ US | 12.0 | 232 | Carvedilol | 50-100mg | 54.3 | 84.9 | 99.0 | 23.0 | ≤ 35% | NA | 41.4 | NA | 48 | NA |
|  |  |  |  | 134 | Placebo | NA |  |  |  |  |  |  |  |  |  |  |
| **Krum 1995^45^** | DB, PC | NA/ US | 3.3 | 33 | Carvedilol | 50mg | 55.5 | 77.6 | 90.0 | 16.3 | ≤ 35% | NA | 26.5 | NA | NA | NA |
|  |  |  |  | 16 | Placebo | NA |  |  |  |  |  |  |  |  |  |  |
| **CIBIS III 2008^46^** | OL, MC | 128/ 20 countries | 5.4 | 505 | Bisoprolol | 10mg | 72.5 | 68.5 | 100.0 | 28.8 | ≤ 35% | NA | 49.0 | 49.0 | 19 | 20.5 |
|  |  |  |  | 505 | Enalapril | 10mg or 20mg |  |  |  |  |  |  |  |  |  |  |
| **CARMEN 2004^47^** | DB, MC | 65/ European | 22.0 | 191 | Carvedilol  +Enalapril | 50mg or 100mg/20mg | 62.3 | 80.7 | 92.0 | NA | < 40% | NA | 67.0 | 51.7 | NA | 14.3 |
|  |  |  |  | 191 | Carvedilol | 50mg or 100mg |  |  |  |  |  |  |  |  |  |  |
|  |  |  |  | 190 | Enalapril | 20mg |  |  |  |  |  |  |  |  |  |  |
| **SHIFT 2010^48^** | DB, MC, PC | 677/ 37 countries | 22.9 | 3241 | Ivabradine | 15mg | 60.4 | 76.4 | 99.0 | 29.0 | ≤ 35% | 74.7 | 67.9 | 56.4 | 42 | 30.4 |
|  |  |  |  | 3264 | Placebo | NA |  |  |  |  |  |  |  |  |  |  |

Abbreviations: DB, double blind; DM, diabetes mellitus; eGFR, estimated glomerular filtration rate; HF, heart failure; LVEF, left ventricular ejection fraction; MC, multi-centre; MI, myocardial infarction; NA, not available; NYHA, New York Heart Association; OL, open label; PC, placebo controlled; QB, quadruple blind; SB, single blind; SC, single centre; US, United States.

**Table S2. Baseline characteristics of included interventions and concomitant therapies reported.**

| **Trial/Author year** | **Intervention class (>50%)** | **Main intervention** | **ACEI (%)** | **ARB (%)** | **BB (%)** | **MRA (%)** | **Digitalis (%)** | **Diuretics (%)** |
| --- | --- | --- | --- | --- | --- | --- | --- | --- |
| **DAPA-HF 2019** | SGLT2+ACEI+BB+MRA | Dapagliflozin | 56 | 28 | 96 | 72 | 19 | 93 |
|  | ACEI+BB+MRA | Placebo | 56 | 27 | 96 | 71 | 19 | 94 |
| **EMPEROR-Reduced 2020** | SGLT2+*ACEI+BB+MRA | Empagliflozin | 71* | 71* | 95 | 70 | NA | NA |
|  | *ACEI+BB+MRA | Placebo | 69* | 69* | 95 | 73 | NA | NA |
| **Empire HF 2019** | SGLT2+*ACEI+BB+MRA | Empagliflozin | 95* | 95* | 96 | 65 | 2 | 65 |
|  | *ACEI+BB+MRA | Placebo | 97* | 97* | 94 | 66 | 2 | 62 |
| **PARADIGM-HF 2014** | ARNI+BB+MRA | LCZ696 | NP | NA | 93 | 54 | 29 | 80 |
|  | ACEI+BB+MRA | Enalapril | NP | NA | 93 | 57 | 31 | 80 |
| **RALES 1999** | ACEI+MRA | Spironolactone | 95 | NA | 11 | NP | 75 | 100 |
|  | ACEI | Placebo | 94 | NA | 10 | NP | 72 | 100 |
| **Vizzardi 2014** | *ACEI+BB+MRA | Spironolactone | 100* | 100* | 97 | 75 | NA | NA |
|  | *ACEI+BB | Placebo | 99* | 99* | 98 | 86 | NA | NA |
| **EMPHASIS-HF 2011** | ACEI+BB+MRA | Eplerenone | 78 | 19 | 87 | NP | 27 | 84 |
|  | ACEI+BB | Placebo | 77 | 19 | 87 | NP | 28 | 86 |
| **Val-HeFT 2001** | ACEI+ARB | Valsartan | 93 | NP | 35 | NA | 67 | 86 |
|  | ACEI | Placebo | 93 | NP | 35 | NA | 68 | 85 |
| **SPICE 2000** | ARB | Candesartan | NP | 11 | 22 | NA | 60 | 76 |
|  | Placebo | Placebo | NP | 10 | 20 | NA | 63 | 71 |
| **CHARM-Alternative 2003** | ARB+BB | Candesartan | NP | NP | 55 | 25 | 45 | 85 |
|  | BB | Placebo | NP | NP | 55 | 23 | 46 | 86 |
| **Mitrovic 2003** | ARB | Candesartan | NP | NP | NA | NA | 75 | 91 |
|  | Placebo | Placebo | NP | NP | NA | NA | 82 | 91 |
| **CHARM-Added 2003** | ACEI+ARB+BB | Candesartan | 100 | NP | 55 | **17** | 58 | 90 |
|  | ACEI+BB | Placebo | 100 | NP | 56 | 17 | 59 | 90 |
| **SOLVD-treat 1991** | ACEI | Enalapril | NP | NA | 8 | NA | 66 | 86 |
|  | Placebo | Placebo | NP | NA | 7 | NA | 68 | 85 |
| **Shettigar 1999** | ACEI | Fosinopril | NP | NA | NP | NA | 42 | 100 |
|  | Placebo | Placebo | NP | NA | NP | NA | 47 | 100 |
| **CASSIS 1995** | ACEI | Spirapril, Enalapril | 5 | NA | NA | NA | 91 | 96 |
|  | Placebo | Placebo | 5 | NA | NA | NA | 91 | 96 |
| **FEST 1995** | ACEI | Fosinopril | 7 | NA | NA | NA | 61 | 100 |
|  | Placebo | Placebo | 7 | NA | NA | NA | 62 | 100 |
| **SOLVD-prevent 1992** | ACEI | Enalapril | NP | NA | 24 | NA | 12 | 16 |
|  | Placebo | Placebo | NP | NA | 24 | NA | 13 | 17 |
| **Brown 1995** | ACEI | Fosinopril | NP | NA | NP | NA | 53 | NA |
|  | Placebo | Placebo | NP | NA | NP | NA | 54 | NA |
| **Colfer 1992** | ACEI | Benazepril | NA | NA | NP | NA | 100 | 100 |
|  | Placebo | Placebo | NA | NA | NP | NA | 100 | 100 |
| **Goldstein 1988** | ACEI | Captopril | 3 | NA | NA | NA | 60 | 78 |
|  | Placebo | Placebo | 11 | NA | NA | NA | 67 | 86 |
| **ELITE I 1997** | ARB | Losartan | NP | NP | 16 | NA | 57 | 74 |
|  | ACEI | Captopril | NP | NP | 17 | NA | 56 | 74 |
| **ELITE II 2000** | ARB | Losartan | 23 | NP | 23 | NA | 50 | 77 |
|  | ACEI | Captopril | 24 | NP | 21 | NA | 50 | 79 |
| **REPLACE 2001** | ARB | Telmisartan | NP | NP | NA | NA | 39 | NA |
|  | ACEI | Enalapril | NP | NP | NA | NA | 39 | NA |
| **Dickstein 1995** | ARB | Losartan | NA | NA | 15 | NA | 66 | 94 |
|  | ACEI | Enalapril | NA | NA | 7 | NA | 59 | 95 |
| **CIBIS I 1994** | ACEI+BB | Bisoprolol | 89 | NA | NA | NA | 57 | 100 |
|  | ACEI | Placebo | 91 | NA | NA | NA | 56 | 100 |
| **RESOLVD 2000** | ACEI+ARB+BB | Metoprolol+(candesartan, enalapril, or candesartan+enalapril) | alone:14;combination:41 | alone:45 | NA | NA | 65 | 84 |
|  | ACEI+ARB | Placebo+(candesartan, enalapril, or candesartan+enalapril) | alone:19;combination:41 | alone:40 | NA | NA | 69 | 83 |
| **Palazzuoli 2005** | ACEI+BB | Carvedilol | 100 | NA | NP | NA | NA | NA |
|  | ACEI | Placebo | 100 | NA | NP | NA | NA | NA |
| **SYMPOXYDEX 2004** | ACEI+BB | Carvedilol | 96 | NA | NP | NA | NA | 100 |
|  | ACEI | Placebo | 96 | NA | NP | NA | NA | 100 |
| **ENECA 2005** | ACEI+BB | Nebivolol | 91 | 5 | NP | NA | 60 | 87 |
|  | ACEI | Placebo | 90 | 7 | NP | NA | 53 | 88 |
| **COPERNICUS 2003** | *ACEI+BB | Carvedilol | 97* | 97* | NP | 19 | 67 | NA |
|  | *ACEI | Placebo | 97* | 97* | NP | 20 | 65 | NA |
| **Dubach 2002** | ACEI+BB | Bisoprolol | 100 | NA | NP | NA | 5 | NA |
|  | ACEI | Placebo | 100 | NA | NP | NA | 9 | NA |
| **Milliano 2002** | ACEI+BB | Metoprolol | 93 | NA | NA | NA | 28 | NA |
|  | ACEI | Placebo | 91 | NA | NA | NA | 36 | NA |
| **Packer 1996** | ACEI+BB | Carvedilol | 95 | NA | NP | NA | 91 | 95 |
|  | ACEI | Placebo | 95 | NA | NP | NA | 90 | 95 |
| **BEST 2001** | ACEI+BB | Bucindolol | 91 | 6 | NP | 3 | 93 | 94 |
|  | ACEI | Placebo | 91 | 7 | NP | 4 | 92 | 94 |
| **MERIT-HF (pilot) 1999** | ACEI+BB | Metoprolol | 95 | NA | NP | NA | 91 | 88 |
|  | ACEI | Placebo | 90 | NA | NP | NA | 90 | 84 |
| **CELICARD 2000** | ACEI+BB | Celiprolol | 80 | NA | NP | NA | 51 | 96 |
|  | ACEI | Placebo | 90 | NA | NP | NA | 66 | 93 |
| **CIBIS-II 1999** | ACEI+BB | Bisoprolol | 96 | NA | NP | NA | 53 | 98 |
|  | ACEI | Placebo | 96 | NA | NP | NA | 51 | 99 |
| **MERIT-HF 1999** | ACEI+BB | Metoprolol | 89 | 7 | NP | NA | 63 | 91 |
|  | ACEI | Placebo | 90 | 6 | NP | NA | 64 | 90 |
| **Cohn 1997** | ACEI+BB | Carvedilol | 94 | NA | NP | NA | 90 | 97 |
|  | ACEI | Placebo | 91 | NA | NP | NA | 89 | 100 |
| **MIC 2000** | ACEI+BB | Metoprolol | 92 | NA | NP | NA | 51 | 71 |
|  | ACEI | Placebo | 91 | NA | NP | NA | 52 | 71 |
| **Sturm 2000** | ACEI+BB | Atenolol | 100 | NA | NP | NA | 100 | 100 |
|  | ACEI | Placebo | 100 | NA | NP | NA | 100 | 90 |
| MOCHA 1996 | ACEI+BB | Carvedilol | 94 | NA | NP | NA | 92 | 96 |
|  | ACEI | Placebo | 94 | NA | NP | NA | 93 | 93 |
| PRECISE 1996 | ACEI+BB | Carvedilol | 96 | NA | NP | NA | 91 | 98 |
|  | ACEI | Placebo | 97 | NA | NP | NA | 88 | 99 |
| Colucci 1996 | ACEI+BB | Carvedilol | 98 | NA | NP | NA | 89 | 92 |
|  | ACEI | Placebo | 98 | NA | NP | NA | 89 | 92 |
| Krum 1995 | ACEI+BB | Carvedilol | 94 | NA | NA | NA | NA | NA |
|  | ACEI | Placebo | 88 | NA | NA | NA | NA | NA |
| **CIBIS III 2008** | BB | Bisoprolol | NP | NA | NP | 14 | 33 | 85 |
|  | ACEI | Enalapril | NP | NA | NP | 12 | 31 | 83 |
| **CARMEN 2004** | ACEI+BB | Carvedilol+Enalapril | NP | NA | 4 | 12 | 47 | 73 |
|  | BB | Carvedilol | NP | NA | 8 | 15 | 44 | 67 |
|  | ACEI | Enalapril | NP | NA | 5 | 13 | 44 | 74 |
| **SHIFT 2010** | IVA+ACEI+BB+MRA | Ivabradine | 79 | 14 | 89 | 61 | 22 | 84 |
|  | ACEI+BB+MRA | Placebo | 78 | 14 | 90 | 59 | 22 | 83 |

* data presented for ACEI/ARB

Abbreviations: ACEI, angiotensin-converting-enzyme inhibitor; ARB, angiotensin-II receptor antagonist; ARNI, angiotensin receptor-neprilysin inhibitor; BB, beta blocker; MRA,mineralocorticoid receptor antagonist; NA, not available; NP, not permitted.

**Table S3. Network meta-analysis for all-cause mortality (A), cardiovascular mortality (B) and hospitalization for heart failure (C):** the point estimate of hazard ratio for intervention vs comparator, 95% credible interval, probability that the intervention is better than the comparator (P value), and heterogeneity (SD).

| **A** | **Comparator** | | | | | | | | | | | |
| --- | --- | --- | --- | --- | --- | --- | --- | --- | --- | --- | --- | --- |
| **Intervention** | **Placebo** | **ACEI** | **ARB** | **BB** | **ACEI+ARB** | **ACEI+BB** | **ACEI+MRA** | **ARB+BB** | **ACEI+BB+MRA** | **ACEI+ARB+BB** | **ARNI+BB+MRA** | **SGLT2i+ACEI+BB+MRA** |
| **ACEI** | | | | | | | | | | | | |
| **Estimate (95% CrI)** | 0.85 (0.65;1.08) |  |  |  |  |  |  |  |  |  |  |  |
| **P value** | 0.79 |  |  |  |  |  |  |  |  |  |  |  |
| **ARB** | | | | | | | | | | | | |
| **Estimate (95% CrI)** | 0.79 (0.49;1.14) | 0.93 (0.64;1.26) |  |  |  |  |  |  |  |  |  |  |
| **P value** | 0.73 | 0.31 |  |  |  |  |  |  |  |  |  |  |
| **BB** | | | | | | | | | | | | |
| **Estimate (95% CrI)** | 0.65 (0.37;1.10) | 0.75 (0.46;1.23) | 0.83 (0.46;1.52) |  |  |  |  |  |  |  |  |  |
| **P value** | 0.88 | 0.74 | 0.46 |  |  |  |  |  |  |  |  |  |
| **ACEI+ARB** | | | | | | | | | | | | |
| **Estimate (95% CrI)** | 0.90 (0.57;1.40) | 1.05 (0.73;1.55) | 1.14 (0.72;1.99) | 1.38 (0.76;2.54) |  |  |  |  |  |  |  |  |
| **P value** | 0.34 | 0.21 | 0.38 | 0.69 |  |  |  |  |  |  |  |  |
| **ACEI+BB** | | | | | | | | | | | | |
| **Estimate (95% CrI)** | 0.59 (0.42;0.78) | 0.69 (0.58;0.81) | 0.75 (0.53;1.10) | 0.90 (0.54;1.48) | 0.65 (0.42;0.96) |  |  |  |  |  |  |  |
| **P value** | 1.00 | 1.00 | 0.88 | 0.31 | 0.96 |  |  |  |  |  |  |  |
| **ACEI+MRA** | | | | | | | | | | | | |
| **Estimate (95% CrI)** | 0.59 (0.35;0.94) | 0.69 (0.45;1.05) | 0.75 (0.45;1.33) | 0.90 (0.48;1.7) | 0.65 (0.37;1.14) | 1.00 (0.65;1.59) |  |  |  |  |  |  |
| **P value** | 0.97 | 0.92 | 0.71 | 0.24 | 0.86 | 0.00 |  |  |  |  |  |  |
| **ARB+BB** | | | | | | | | | | | | |
| **Estimate (95% CrI)** | 0.57 (0.28;1.12) | 0.66 (0.35;1.26) | 0.73 (0.36;1.54) | 0.88 (0.58;1.34) | 0.64 (0.30;1.32) | 0.97 (0.50;1.89) | 0.97 (0.45;2.10) |  |  |  |  |  |
| **P value** | 0.89 | 0.79 | 0.61 | 0.44 | 0.76 | 0.07 | 0.06 |  |  |  |  |  |
| **ACEI+BB+MRA** | | | | | | | | | | | | |
| **Estimate (95% CrI)** | 0.48 (0.29;0.78) | 0.56 (0.36;0.86) | 0.61 (0.36;1.09) | 0.74 (0.39;1.39) | 0.53 (0.30;0.92) | 0.82 (0.56;1.23) | 0.82 (0.44;1.50) | 0.85 (0.40;1.83) |  |  |  |  |
| **P value** | 1.00 | 0.99 | 0.92 | 0.65 | 0.97 | 0.67 | 0.47 | 0.32 |  |  |  |  |
| **ACEI+ARB+BB** | | | | | | | | | | | | |
| **Estimate (95% CrI)** | 0.51 (0.30;0.79) | 0.60 (0.39;0.87) | 0.64 (0.39;1.10) | 0.78 (0.41;1.42) | 0.56 (0.33;0.89) | 0.86 (0.58;1.25) | 0.86 (0.47;1.53) | 0.90 (0.42;1.87) | 1.05 (0.60;1.78) |  |  |  |
| **P value** | 0.99 | 0.99 | 0.91 | 0.57 | 0.98 | 0.55 | 0.36 | 0.22 | 0.14 |  |  |  |
| **ARNI+BB+MRA** | | | | | | | | | | | | |
| **Estimate (95% CrI)** | 0.40 (0.21;0.76) | 0.48 (0.26;0.85) | 0.51 (0.27;1.05) | 0.62 (0.29;1.31) | 0.45 (0.22;0.88) | 0.69 (0.40;1.22) | 0.69 (0.33;1.43) | 0.71 (0.30;1.69) | 0.84 (0.56;1.26) | 0.80 (0.41;1.62) |  |  |
| **P value** | 0.99 | 0.99 | 0.94 | 0.78 | 0.98 | 0.80 | 0.68 | 0.55 | 0.59 | 0.46 |  |  |
| **SGLT2i+ACEI+BB+MRA** | | | | | | | | | | | | |
| **Estimate (95% CrI)** | 0.42 (0.23;0.75) | 0.50 (0.29;0.83) | 0.53 (0.30;1.04) | 0.65 (0.32;1.31) | 0.47 (0.24;0.87) | 0.72 (0.44;1.19) | 0.72 (0.36;1.41) | 0.74 (0.33;1.69) | 0.88 (0.65;1.18) | 0.83 (0.45;1.6) | 1.04 (0.63;1.71) |  |
| **P value** | 1.00 | 0.99 | 0.95 | 0.77 | 0.98 | 0.81 | 0.66 | 0.51 | 0.6 | 0.41 | 0.11 |  |
| **IVA+ACEI+BB+MRA** | | | | | | | | | | | | |
| **Estimate (95% CrI)** | 0.44 (0.23;0.82) | 0.51 (0.28;0.92) | 0.55 (0.29;1.14) | 0.67 (0.31;1.42) | 0.48 (0.24;0.96) | 0.75 (0.43;1.33) | 0.74 (0.35;1.55) | 0.77 (0.32;1.83) | 0.91 (0.61;1.37) | 0.87 (0.44;1.76) | 1.08 (0.61;1.91) | 1.04 (0.63;1.72) |
| **P value** | 0.99 | 0.97 | 0.91 | 0.70 | 0.96 | 0.68 | 0.56 | 0.43 | 0.34 | 0.30 | 0.19 | 0.11 |
| Heterogeneity: SD 0.17, 95% CrI 0.05-0.35 | | | | | | | | | | | | |

| **B** | **Comparator** | | | | | | | | | | | |
| --- | --- | --- | --- | --- | --- | --- | --- | --- | --- | --- | --- | --- |
| **Intervention** | **Placebo** | **ACEI** | **ARB** | **BB** | **ACEI+ARB** | **ACEI+BB** | **ACEI+MRA** | **ARB+BB** | **ACEI+BB+MRA** | **ACEI+ARB+BB** | **ARNI+BB+MRA** | **SGLT2i+ACEI+BB+MRA** |
| **ACEI** | | | | | | | | | | | | |
| **Estimate (95% CrI)** | 0.85 (0.57;1.25) |  |  |  |  |  |  |  |  |  |  |  |
| **P value** | 0.58 |  |  |  |  |  |  |  |  |  |  |  |
| **ARB** | | | | | | | | | | | | |
| **Estimate (95% CrI)** | 0.76 (0.40;1.30) | 1.31 (0.58;2.65) |  |  |  |  |  |  |  |  |  |  |
| **P value** | 0.62 | 0.50 |  |  |  |  |  |  |  |  |  |  |
| **BB** | | | | | | | | | | | | |
| **Estimate (95% CrI)** | 0.74 (0.28;1.85) | 1.26 (0.44;3.47) | 0.96 (0.37;2.58) |  |  |  |  |  |  |  |  |  |
| **P value** | 0.47 | 0.33 | 0.06 |  |  |  |  |  |  |  |  |  |
| **ACEI+ARB** | | | | | | | | | | | | |
| **Estimate (95% CrI)** | 0.86 (0.41;1.77) | 1.47 (0.63;3.48) | 1.12 (0.57;2.59) | 1.17 (0.43;3.38) |  |  |  |  |  |  |  |  |
| **P value** | 0.30 | 0.62 | 0.23 | 0.22 |  |  |  |  |  |  |  |  |
| **ACEI+BB** | | | | | | | | | | | | |
| **Estimate (95% CrI)** | 0.52 (0.31;0.80) | 0.90 (0.45;1.68) | 0.69 (0.42;1.19) | 0.72 (0.31;1.69) | 0.62 (0.31;1.13) |  |  |  |  |  |  |  |
| **P value** | 0.99 | 0.23 | 0.84 | 0.56 | 0.85 |  |  |  |  |  |  |  |
| **ACEI+MRA** | | | | | | | | | | | | |
| **Estimate (95% CrI)** | 0.59 (0.28;1.21) | 1.71 (0.85;3.57) | 0.77 (0.39;1.77) | 0.8 (0.30;2.32) | 0.69 (0.30;1.62) | 1.12 (0.61;2.25) |  |  |  |  |  |  |
| **P value** | 0.85 | 0.86 | 0.49 | 0.32 | 0.60 | 0.25 |  |  |  |  |  |  |
| **ARB+BB** | | | | | | | | | | | | |
| **Estimate (95% CrI)** | 0.63 (0.20;1.87) | 1.07 (0.32;3.49) | 0.82 (0.27;2.67) | 0.85 (0.46;1.56) | 0.74 (0.22;2.40) | 1.19 (0.42;3.39) | 1.06 (0.30;3.55) |  |  |  |  |  |
| **P value** | 0.58 | 0.09 | 0.25 | 0.38 | 0.36 | 0.24 | 0.06 |  |  |  |  |  |
| **ACEI+BB+MRA** | | | | | | | | | | | | |
| **Estimate (95% CrI)** | 0.37 (0.16;0.71) | 0.63 (0.25;1.39) | 0.48 (0.23;1.01) | 0.50 (0.18;1.38) | 0.43 (0.17;0.94) | 0.70 (0.38;1.18) | 0.63 (0.23;1.39) | 0.58 (0.17;1.79) |  |  |  |  |
| **P value** | 0.99 | 0.70 | 0.94 | 0.81 | 0.94 | 0.78 | 0.69 | 0.63 |  |  |  |  |
| **ACEI+ARB+BB** | | | | | | | | | | | | |
| **Estimate (95% CrI)** | 0.44 (0.20;0.91) | 0.76 (0.30;1.79) | 0.58 (0.27;1.32) | 0.61 (0.22;1.7) | 0.52 (0.21;1.22) | 0.85 (0.46;1.54) | 0.76 (0.29;1.81) | 0.70 (0.21;2.23) | 1.21 (0.56;2.87) |  |  |  |
| **P value** | 0.96 | 0.44 | 0.82 | 0.66 | 0.85 | 0.40 | 0.44 | 0.44 | 0.33 |  |  |  |
| **ARNI+BB+MRA** | | | | | | | | | | | | |
| **Estimate (95% CrI)** | 0.29 (0.10;0.69) | 0.50 (0.16;1.33) | 0.39 (0.15;0.98) | 0.40 (0.12;1.26) | 0.34 (0.11;0.89) | 0.56 (0.24;1.20) | 0.50 (0.15;1.33) | 0.46 (0.12;1.61) | 0.80 (0.44;1.44) | 0.66 (0.23;1.73) |  |  |
| **P value** | 0.99 | 0.80 | 0.95 | 0.87 | 0.96 | 0.84 | 0.79 | 0.75 | 0.54 | 0.57 |  |  |
| **SGLT2i+ACEI+BB+MRA** | | | | | | | | | | | | |
| **Estimate (95% CrI)** | 0.32 (0.12;0.70) | 0.55 (0.19;1.33) | 0.42 (0.17;0.98) | 0.44 (0.14;1.29) | 0.38 (0.13;0.89) | 0.61 (0.29;1.19) | 0.54 (0.18;1.33) | 0.50 (0.14;1.67) | 0.87 (0.57;1.34) | 0.72 (0.27;1.74) | 1.09 (0.52;2.25) |  |
| **P value** | 0.99 | 0.77 | 0.95 | 0.86 | 0.96 | 0.83 | 0.77 | 0.72 | 0.46 | 0.50 | 0.16 |  |
| **IVA+ACEI+BB+MRA** | | | | | | | | | | | | |
| **Estimate (95% CrI)** | 0.34 (0.12;0.80) | 0.58 (0.19;1.53) | 0.44 (0.17;1.12) | 0.46 (0.14;1.45) | 0.40 (0.13;1.03) | 0.64 (0.27;1.39) | 0.57 (0.18;1.53) | 0.53 (0.14;1.85) | 0.92 (0.50;1.66) | 0.76 (0.26;2.00) | 1.14 (0.49;2.63) | 1.05 (0.50;2.21) |
| **P value** | 0.98 | 0.69 | 0.91 | 0.80 | 0.92 | 0.71 | 0.68 | 0.66 | 0.22 | 0.39 | 0.23 | 0.10 |
| Heterogeneity: SD 0.26, 95% CrI 0.07-0.53 | | | | | | | | | | | | |

| **C** | **Comparator** | | | | | | | | | | | |
| --- | --- | --- | --- | --- | --- | --- | --- | --- | --- | --- | --- | --- |
| **Intervention** | **Placebo** | **ACEI** | **ARB** | **BB** | **ACEI+ARB** | **ACEI+BB** | **ACEI+MRA** | **ARB+BB** | **ACEI+BB+MRA** | **ACEI+ARB+BB** | **ARNI+BB+MRA** | **SGLT2i+ACEI+BB+MRA** |
| **ACEI** | | | | | | | | | | | | |
| **Estimate (95% CrI)** | 0.60 (0.43;0.75) |  |  |  |  |  |  |  |  |  |  |  |
| **P value** | 1.00 |  |  |  |  |  |  |  |  |  |  |  |
| **ARB** | | | | | | | | | | | | |
| **Estimate (95% CrI)** | 0.58 (0.38;0.84) | 0.95 (0.70;1.39) |  |  |  |  |  |  |  |  |  |  |
| **P value** | 0.99 | 0.24 |  |  |  |  |  |  |  |  |  |  |
| **BB** | | | | | | | | | | | | |
| **Estimate (95% CrI)** | 0.81 (0.43;1.37) | 1.34 (0.81;2.10) | 1.4 (0.74;2.46) |  |  |  |  |  |  |  |  |  |
| **P value** | 0.51 | 0.77 | 0.73 |  |  |  |  |  |  |  |  |  |
| **ACEI+ARB** | | | | | | | | | | | | |
| **Estimate (95% CrI)** | 0.39 (0.19;0.58) | 0.65 (0.37;0.89) | 0.68 (0.34;1.05) | 0.48 (0.24;0.86) |  |  |  |  |  |  |  |  |
| **P value** | 1.00 | 0.95 | 0.82 | 0.98 |  |  |  |  |  |  |  |  |
| **ACEI+BB** |  |  |  |  |  |  |  |  |  |  |  |  |
| **Estimate (95% CrI)** | 0.42 (0.25;0.58) | 0.70 (0.51;0.86) | 0.74 (0.44;1.04) | 0.53 (0.31;0.86) | 1.08 (0.71;1.80) |  |  |  |  |  |  |  |
| **P value** | 1.00 | 0.99 | 0.83 | 0.99 | 0.25 |  |  |  |  |  |  |  |
| **ACEI+MRA** | | | | | | | | | | | | |
| **Estimate (95% CrI)** | 0.42 (0.23;0.67) | 0.69 (0.44;1.08) | 0.72 (0.39;1.23) | 0.51 (0.27;1.02) | 1.06 (0.63;2.25) | 0.98 (0.61;1.75) |  |  |  |  |  |  |
| **P value** | 1.00 | 0.90 | 0.73 | 0.95 | 0.13 | 0.07 |  |  |  |  |  |  |
| **ARB+BB** | | | | | | | | | | | | |
| **Estimate (95% CrI)** | 0.56 (0.25;1.08) | 0.92 (0.46;1.72) | 0.97 (0.43;1.94) | 0.69 (0.44;1.09) | 1.42 (0.69;3.3) | 1.30 (0.68;2.59) | 1.33 (0.58;2.86) |  |  |  |  |  |
| **P value** | 0.87 | 0.18 | 0.06 | 0.88 | 0.62 | 0.55 | 0.50 |  |  |  |  |  |
| **ACEI+BB+MRA** | | | | | | | | | | | | |
| **Estimate (95% CrI)** | 0.27 (0.13;0.44) | 0.44 (0.24;0.7) | 0.46 (0.22;0.79) | 0.33 (0.16;0.64) | 0.68 (0.37;1.37) | 0.63 (0.40;1.00) | 0.65 (0.30;1.23) | 0.47(0.20;1.1) |  |  |  |  |
| **P value** | 1.00 | 1.00 | 0.98 | 1.00 | 0.76 | 0.95 | 0.77 | 0.92 |  |  |  |  |
| **ACEI+ARB+BB** | | | | | | | | | | | | |
| **Estimate (95% CrI)** | 0.41 (0.24;0.69) | 0.68 (0.45;1.13) | 0.71 (0.41;1.28) | 0.51 (0.28;1.04) | 1.05 (0.66;2.16) | 0.96 (0.69;1.65) | 1 (0.55;1.99) | 0.73(0.35;1.80) | 1.53 (0.91;3.25) |  |  |  |
| **P value** | 1.00 | 0.90 | 0.76 | 0.96 | 0.11 | 0.12 | 0.01 | 0.54 | 0.81 |  |  |  |
| **ARNI+BB+MRA** | | | | | | | | | | | | |
| **Estimate (95% CrI)** | 0.22 (0.09;0.41) | 0.36 (0.17;0.66) | 0.37 (0.16;0.73) | 0.27 (0.11;0.59) | 0.55 (0.26;1.28) | 0.51 (0.27;0.96) | 0.52 (0.21;1.13) | 0.38(0.15;0.99) | 0.81 (0.52;1.26) | 0.52 (0.22;1.02) |  |  |
| **P value** | 1.00 | 1.00 | 0.99 | 1.00 | 0.86 | 0.96 | 0.87 | 0.95 | 0.65 | 0.90 |  |  |
| **SGLT2i+ACEI+BB+MRA** | | | | | | | | | | | | |
| **Estimate (95% CrI)** | 0.19 (0.09;0.35) | 0.32 (0.16;0.56) | 0.33 (0.15;0.62) | 0.24 (0.11;0.49) | 0.48 (0.25;1.08) | 0.45 (0.26;0.80) | 0.46 (0.20;0.95) | 0.34(0.14;0.85) | 0.71 (0.53;1.00) | 0.46 (0.21;0.86) | 0.89 (0.52;1.60) |  |
| **P value** | 1.00 | 1.00 | 1.00 | 1.00 | 0.95 | 0.99 | 0.95 | 0.98 | 0.96 | 0.97 | 0.31 |  |
| **IVA+ACEI+BB+MRA** | | | | | | | | | | | | |
| **Estimate (95% CrI)** | 0.20 (0.08;0.38) | 0.33 (0.16;0.61) | 0.35 (0.14;0.68) | 0.25 (0.11;0.54) | 0.51 (0.24;1.19) | 0.47 (0.25;0.89) | 0.49 (0.20;1.04) | 0.35(0.14;0.92) | 0.75 (0.48;1.17) | 0.49 (0.20;0.94) | 0.93 (0.49;1.78) | 1.05 (0.59;1.78) |
| **P value** | 1.00 | 1.00 | 0.99 | 1.00 | 0.91 | 0.98 | 0.91 | 0.97 | 0.80 | 0.93 | 0.17 | 0.12 |
| Heterogeneity: SD 0.16, 95% CrI 0.01-0.47 | | | | | | | | | | | | |

**Supplemental references**

1. McMurray JJV, Solomon SD, Inzucchi SE, Køber L, Kosiborod MN. Dapagliflozin in Patients with Heart Failure and Reduced Ejection Fraction. *New Engl J Med.* 2019;381:1995-2008.

2. Packer M, Anker SD, Butler J, Filippatos G, Pocock SJ. Cardiovascular and Renal Outcomes with Empagliflozin in Heart Failure. *New Engl J Med.* 2020;383:1413-1424.

3. Jensen J, Omar M, Kistorp C, Poulsen MK, Tuxen C. Twelve weeks of treatment with empagliflozin in patients with heart failure and reduced ejection fraction: A double-blinded, randomized, and placebo-controlled trial. *Am Heart J.* 2020;228:47-56.

4. McMurray JJV, Packer M, Desai AS, Gong JJ, Lefkowitz MP. Angiotensin-Neprilysin Inhibition versus Enalapril in Heart Failure. *New Engl J Med.* 2014;371:993-1004.

5. Pitt B, Zannad F, Remme WJ, Cody R, Castaigne A. The effect of spironolactone on morbidity and mortality in patients with severe heart failure. *New Engl J Med.* 1999;341:709-717.

6. Vizzardi E, Nodari S, Caretta G, D'Aloia A, Pezzali N. Effects of Spironolactone on Long-term Mortality and Morbidity in Patients With Heart Failure and Mild or No Symptoms. *Am J Med Sci.* 2014;347:271-276.

7. Zannad F, McMurray JJV, Krum H, van Veldhuisen DJ, Swedberg K. Eplerenone in Patients with Systolic Heart Failure and Mild Symptoms. *New Engl J Med.* 2011;364:11-21.

8. Cohn JN, Tognoni G, Valsartan Heart Failure Trial I. A randomized trial of the angiotensin-receptor blocker valsartan in chronic heart failure. *New Engl J Med.* 2001;345:1667-1675.

9. Granger CB, Ertl G, Kuch J, Maggioni AP, McMurray J. Randomized trial of candesartan cilexetil in the treatment of patients with congestive heart failure and a history of intolerance to angiotensin-converting enzyme inhibitors. *Am Heart J.* 2000;139:609-617.

10. Granger CB, McMurray JJV, Yusuf S, Held P, Michelson EL. Effects of candesartan in patients with chronic heart failure and reduced left-ventricular systolic function intolerant to angiotensin-converting-enzyme inhibitors: the CHARM-Alternative trial. *Lancet.* 2003;362:772-776.

11. Mitrovic V, Willenbrock R, Miric M, Seferovic P, Spinar J. Acute and 3-month treatment effects of candesartan cilexetil on hemodynamics, neurohormones, and clinical symptoms in patients with congestive heart failure. *Am Heart J.* 2003;145:9.

12. McMurray JJV, Ostergren J, Swedberg K, Granger CB, Held P. Effects of candesartan in patients with chronic heart failure and reduced left-ventricular systolic function taking angiotensin-converting-enzyme inhibitors: the CHARM-Added trial. *Lancet.* 2003;362:767-771.

13. Yusuf S. Effect of enalapril on survival in patients with reduced left-ventricular ejection fractions and congestive-heart-failure. *New Engl J Med* 1991;325:293-302.

14. Shettigar U, Hare T, Gelperin K, Ilgenfritz JP, Deitchman D, Blumenthal M. Effects of fosinopril on exercise tolerance, symptoms, and clinical outcomes in patients with decompensated heart failure. *Congest Heart Fail.* 1999;5:27-34.

15. Widimsky J, Kremer HJ, Jerie P, Uhlir O. Czech and slovak spirapril intervention study (CASSIS) - a randomized, placebo and active-controlled, double-blind multicenter trial in patients with congestive-heart-failure. *Eur J Clin Pharmacol.* 1995;49:95-102.

16. Erhardt L, MacLean A, Ilgenfritz J, Gelperin K. Fosinopril attenuates clinical deterioration and improves exercise tolerance in patients with heart failure. *Eur Heart J.* 1995;16:1892-1899.

17. Nicklas JM, Pitt B, Timmis G, Breneman G, Jafri SM. Effect of enalapril on mortality and the development of heart-failure in asymptomatic patients with reduced left-ventricular ejection fractions. *New Engl J Med.* 1992;327:685-691.

18. Brown EJ, Chew PH, Maclean A, Gelperin K, Ilgenfritz JP, Blumenthal M. Effects of fosinopril on exercise tolerance and clinical deterioration in patients with chronic congestive-heart-failure not taking digitalis. *Am J Cardiol.* 1995;75:596-600.

19. Colfer HT, Ribner HS, Gradman A, Hughes CV, Kapoor A, Laidlaw JC. Effects of once-daily benazepril therapy on exercise tolerance and manifestations of chronic congestive-heart-failure. *Am J Cardiol.* 1992;70:354-358.

20. Goldstein S. Comparative effects of therapy with captopril and digoxin in patients with mild to moderate heart-failure. *JAMA* 1988;259:539-544.

21. Pitt B, Segal R, Martinez FA, Meurers G, Cowley AJ. Randomised trial of losartan versus captopril in patients over 65 with heart failure (Evaluation of Losartan in the Elderly Study, ELITE). *Lancet.* 1997;349:747-752.

22. Pitt B, Poole-Wilson PA, Segal R, Martinez FA, Dickstein K. Effect of losartan compared with captopril on mortality in patients with symptomatic heart failure: randomised trial - the Losartan Heart Failure Survival Study ELITE II. *Lancet.* 2000;355:1582-1587.

23. Dunselman P. Effects of the replacement of the angiotensin converting enzyme inhibitor enalapril by the angiotensin II receptor blocker telmisartan in patients with congestive heart failure - The replacement of angiotensin converting enzyme inhibition (REPLACE) investigators. *Int J Cardiol.* 2001;77:131-138.

24. Dickstein K, Chang P, Willenheimer R, Haunso S, Remes J. Comparison of the effects of losartan and enalapril on clinical status and exercise performance in patients with moderate or severe chronic heart-failure. *J Am Coll Cardiol.* 1995;26:438-445.

25. Lechat P, Jaillon P, Fontaine ML, Chanton E, Mesenge C. A randomized trial of beta-blockade in heart-failure - The Cardiac-Insufficiency Bisoprolol Study (CIBIS). *Circulation.* 1994;90:1765-1773.

26. Cirillo W, Decanini R, Coelho OR, Avezum A, Peixoto MSP. Effects of metoprolol CR in patients with ischemic and dilated cardiomyopathy - The randomized evaluation of strategies for left ventricular dysfunction pilot study. *Circulation.* 2000;101:378-384.

27. Palazzuoli A, Quatrini I, Vecchiato L, Calabria P, Gennari L. Left ventricular diastolic function improvement by carvedilol therapy in advanced heart failure. *J Cardiovasc Pharmacol.* 2005;45:563-568.

28. Cohen Solal A, Jondeau G, Beauvais F, Berdeaux A. Beneficial effects of carvedilol on angiotensin-converting enzyme activity and renin plasma levels in patients with chronic heart failure. *Eur J Heart Fail.* 2004;6:463-466.

29. Edes I, Gasior Z, Wita K. Effects of nebivolol on left ventricular function in elderly patients with chronic heart failure: results of the ENECA study. *Eur J Heart Fail.* 2005;7:631-639.

30. Krum H, Roecker EB, Mohacsi P, Rouleau JL, Tendera M. Effects of initiating carvedilol in patients with severe chronic heart failure - Results from the COPERNICUS study. *JAMA.* 2003;289:712-718.

31. Dubach P, Myers J, Bonetti P, Schertler T, Froelicher V. Effects of bisoprolol fumarate on left ventricular size, function, and exercise capacity in patients with heart failure: Analysis with magnetic resonance myocardial tagging. *Am Heart J.* 2002;143:676-683.

32. de Milliano PAR, de Groot AC, Tijssen JGP, van Eck-Smit BLF, Van Zwieten PA, Lie KI. Beneficial effects of metoprolol on myocardial sympathetic function: Evidence from a randomized, placebo-controlled study in patients with congestive heart failure. *Am Heart J.* 2002;144:E3.

33. Packer M, Bristow MR, Cohn JN, Colucci WS, Fowler MB. The effect of carvedilol on morbidity and mortality in patients with chronic heart failure. *New Engl J Med.* 1996;334:1349-1355.

34. Eichhorn E, Domanski M, Krause-Steinrauf H, Anderson J, Boardman K. A trial of the beta-blocker bucindolol in patients with advanced chronic heart failure. *New Engl J Med.* 2001;344:1659-1667.

35. Goldstein S, Kennedy HL, Hall C, Anderson JL, Gheorghiade M. Metoprolol CR/XL in patients with heart failure: A pilot study examining the tolerability, safety, and effect on left ventricular ejection fraction. *Am Heart J.* 1999;138:1158-1165.

36. Witchitz S, Cohen-Solal A, Dartois N, Weisslinger N, Juste K. Treatment of heart failure with Celiprolol, a cardioselective beta blocker with beta-2 agonist vasodilatory properties. *Am J Cardiol.* 2000;85:1467-1471.

37. Lechat P, Brunhuber KW, Hofmann R, Kuhn P, Nesser HJ. The Cardiac Insufficiency Bisoprolol Study II (CIBIS-II): a randomised trial. *Lancet.* 1999;353:9-13.

38. Hjalmarson A, Goldstein S, Fagerberg B, Wedel H, Waagstein F. Effect of metoprolol CR XL in chronic heart failure: Metoprolol CR XL Randomised Intervention Trial in Congestive Heart Failure (MERIT-HF). *Lancet.* 1999;353:2001-2007.

39. Cohn JN, Fowler MB, Bristow MR, Colucci WS, Gilbert EM. Safety and efficacy of carvedilol in severe heart failure. The U.S. Carvedilol Heart Failure Study Group. *J Card Fail.* 1997;3:173-179.

40. Genth-Zotz S, Zotz RJ, Sigmund M, Hanrath P, Hartmann D. MIC trial: metoprolol in patients with mild to moderate heart failure: effects on ventricular function and cardiopulmonary exercise testing. *Eur J Heart Fail.* 2000;2:175-181.

41. Sturm B, Pacher R, Strametz-Juranek J, Berger R, Frey B, Stanek B. Effect of beta 1 blockade with atenolol on progression of heart failure in patients pretreated with high-dose enalapril. *Eur J Heart Fail.* 2000;2:407-412.

42. Bristow MR, Gilbert EM, Abraham WT, Adams KF, Fowler MB. Carvedilol produces dose-related improvements in left ventricular function and survival in subjects with chronic heart failure. *Circulation.* 1996;94:2807-2816.

43. Packer M, Colucci WS, SacknerBernstein JD, Liang CS, Goldscher DA. Double-blind, placebo-controlled study of the effects of carvedilol in patients with moderate to severe heart failure - The PRECISE trial. *Circulation.* 1996;94:2793-2799.

44. Colucci WS, Packer M, Bristow MR, Gilbert EM, Cohn JN. Carvedilol inhibits clinical progression in patients with mild symptoms of heart failure. *Circulation.* 1996;94:2800-2806.

45. Krum H, Sacknerbernstein JD, Goldsmith RL, Kukin ML, Schwartz B. Double-blind, placebo-controlled study of the long-term efficacy of carvedilol in patients with severe chronic heart-failure. *Circulation.* 1995;92:1499-1506.

46. Dobre D, van Veldhuisen DJ, Goulder MA, Krum H, Willenheimer R. Clinical effects of initial 6 months monotherapy with bisoprolol versus enalapril in the treatment of patients with mild to moderate chronic heart failure. Data from the CIBIS III trial. *Cardiovasc Drugs Ther* 2008;22:399-405.

47. Komajda M, Lutiger B, Madeira H, Thygesen K, Bobbio M. Tolerability of carvedilol and ACE-inhibition in mild heart failure. Results of CARMEN (Carvedilol ACE-Inhibitor Remodelling Mild CHF EvaluatioN). *Eur J Heart Fail.* 2004;6:467-475.

48. Swedberg K, Komajda M, Böhm M, Borer JS, Ford I. Ivabradine and outcomes in chronic heart failure (SHIFT): a randomised placebo-controlled study. *Lancet.* 2010;376:875-885.
